# Whole Proteome Analyses on *Ruminiclostridium cellulolyticum* Show a Modulation of the Cellulolysis Machinery in Response to Cellulosic Materials with Subtle Differences in Chemical and Structural Properties

**DOI:** 10.1371/journal.pone.0170524

**Published:** 2017-01-23

**Authors:** Nelly Badalato, Alain Guillot, Victor Sabarly, Marc Dubois, Nina Pourette, Bruno Pontoire, Paul Robert, Arnaud Bridier, Véronique Monnet, Diana Z. Sousa, Sylvie Durand, Laurent Mazéas, Alain Buléon, Théodore Bouchez, Gérard Mortha, Ariane Bize

**Affiliations:** 1 UR HBAN, Irstea, Antony, France; 2 UMR 1319 MICALIS, PAPPSO, INRA, Jouy-en-Josas, France; 3 Omics Services, Paris, France; 4 UR 1268 BIA, INRA, Nantes, France; 5 Centre of Biological Engineering, University of Minho, Braga, Portugal; 6 Laboratory of Microbiology, Wageningen University, Wageningen, The Netherlands; 7 LGP2, UMR CNRS 5518, Grenoble INP-Pagora, Saint Martin d'Hères, France; National Renewable Energy Laboratory, UNITED STATES

## Abstract

Lignocellulosic materials from municipal solid waste emerge as attractive resources for anaerobic digestion biorefinery. To increase the knowledge required for establishing efficient bioprocesses, dynamics of batch fermentation by the cellulolytic bacterium *Ruminiclostridium cellulolyticum* were compared using three cellulosic materials, paper handkerchief, cotton discs and Whatman filter paper. Fermentation of paper handkerchief occurred the fastest and resulted in a specific metabolic profile: it resulted in the lowest acetate-to-lactate and acetate-to-ethanol ratios. By shotgun proteomic analyses of paper handkerchief and Whatman paper incubations, 151 proteins with significantly different levels were detected, including 20 of the 65 cellulosomal components, 8 non-cellulosomal CAZymes and 44 distinct extracytoplasmic proteins. Consistent with the specific metabolic profile observed, many enzymes from the central carbon catabolic pathways had higher levels in paper handkerchief incubations. Among the quantified CAZymes and cellulosomal components, 10 endoglucanases mainly from the GH9 families and 7 other cellulosomal subunits had lower levels in paper handkerchief incubations. An in-depth characterization of the materials used showed that the lower levels of endoglucanases in paper handkerchief incubations could hypothetically result from its lower crystallinity index (50%) and degree of polymerization (970). By contrast, the higher hemicellulose rate in paper handkerchief (13.87%) did not result in the enhanced expression of enzyme with xylanase as primary activity, including enzymes from the “*xyl-doc*” cluster. It suggests the absence, in this material, of molecular structures that specifically lead to xylanase induction. The integrated approach developed in this work shows that subtle differences among cellulosic materials regarding chemical and structural characteristics have significant effects on expressed bacterial functions, in particular the cellulolysis machinery, resulting in different metabolic patterns and degradation dynamics.

## Introduction

Conversion of cellulose during the degradation of biomass residues and agricultural waste and products has been extensively studied in the context of biofuel production [1–3]. Other sources of lignocellulosic materials, such as waste, are currently emerging as attractive options for biorefinery based on anaerobic digestion [4–6]. The cellulosic fraction in municipal solid waste (MSW) accounts for up to 50% weight in developed countries. Using this resource for biofuel or synthon production can potentially cuts down emissions of greenhouse gases while improving resource efficiency. In contrast with native biomass, this lignocellulosic fraction mainly contains diverse manufactured products with heterogeneous properties such as sanitary textiles, papers, or cardboards.

The optimal strategies to efficiently recover energy or added-value molecules from these specific waste materials are not fully established yet. Approaches relying on fermentation by pure strain cultures, similar to current bioprocesses for bioethanol or biofuel production, could be considered, such as various processes based on fermentation by yeasts [6] or consolidated bioprocessing with various microorganisms such as the bacterium *Lachnoclostridium phytofermentans* or the fungus *Trichoderma reesei* [7, 8]). Alternatively, bioprocesses based on the action of complex microbial communities, such as those classically used for organic waste treatment and valorization (e.g. methanization) could also be invaluable options [5, 9].

Characterization and understanding of the fermentation process of lignocellulosic manufactured materials are needed to establish the scientific bases required for the development of bioprocesses efficiently exploiting their potential. In this respect, a limited number of such studies have been published so far [9–12]. The present work focuses on three cellulosic materials containing no lignin, cotton discs, paper handkerchief and Whatman filter paper, which will be referred as “Cotton”, “Tissue” and “Whatman paper”, respectively. These substrates are rather homogeneous compared to the variety of lignocellulosic waste materials and their bioconversion has been only little studied so far [9–12].

To characterize their anaerobic fermentation dynamics and mechanisms in simple model conditions, *Ruminiclostridium cellulolyticum*, formerly known as *Clostridium cellulolyticum*, was selected as model species. Although the wild-type strain does not produce high concentrations of ethanol nor other biofuel or platform molecules (acetate being the main metabolic end-product), the species is currently considered as a model organism for consolidated bioprocessing through metabolic engineering as exemplified by a recently engineered strain producing isobutanol directly from cellulose [13]. Moreover, closely related members of *R*. *cellulolyticum* have been detected in anaerobic digesters treating waste with high cellulose content [14] and the species has recently been shown to improve wheat straw methanization by bioaugmentation [15]. Finally, the wild-type *R*. *cellulolyticum* bacterium is an important biological model of mesophilic anaerobic cellulolytic bacterium, so that a robust knowledge framework is available for data interpretation including knowledge on the cellulolysis machinery and on its metabolism upon growth on cellulose and its derivatives [16–18]. In particular, detailed studies of its metabolism upon growth on cellobiose *versus* cellulose showed key metabolic nodes in the central metabolic pathways [18, 19]. Its cellulolysis machinery relies both on cellulosomal proteins and non-cellulosomal secreted enzymes [20]. Cellulosomes are complex extracellular muti-enzyme machineries produced by numerous cellulolytic microorganisms. The modulation of *R*. *cellulolyticum* cellulosome composition at the protein level according to the carbohydrate growth substrate has been described in details by targeted approaches [16, 17]. Recently, a transcriptomic and proteomic study of *R*. *cellulolyticum* grown on a variety of substrates (glucose, xylose, cellobiose, cellulose, xylan or corn stover) showed that core cellulases are regulated by carbon catabolite repression, while most of the accessory CAZymes and their associated transporters are regulated by the Two-Component Systems [21].

To achieve a global insight into the bioconversion dynamics and mechanisms of the three studied cellulosic materials by *R*. *cellulolyticum*, a combined approach was developed. The fermentation dynamics was monitored in batch microcosms and the biodegradation mechanisms were analyzed at the protein level for Tissue and Whatman Paper through label-free quantitative shotgun proteomics. The in-depth characterization of the three materials was moreover conducted to elaborate precise hypotheses regarding the origin of the differences in metabolic end-product concentrations and in protein levels. The present work is, to our knowledge, the first global shotgun proteomic study of *R*. *cellulolyticum*. The obtained data provide evidence that Tissue is degraded the fastest by *R*. *cellulolyticum* and is associated to a distinct metabolic pattern compared to both other materials. When comparing Tissue and Whatman Paper, the data show a clear influence of the substrates, even though they are rather similar, on protein levels from the cellulolysis machinery and the central carbon metabolism. Based on the material characteristics, it is postulated that the crystallinity rate and the degree of polymerization had a preponderant influence on the cellulosome composition here, compared to the hemicellulose content.

## Materials and Methods

### Bacterial strain and culture conditions

*R*. *cellulolyticum* H10 ATCC 35319 (DSM 5812) was grown anaerobically at 37°C, as indicated on ATCC website (www.lgcstandards-atcc.org/Products/Cells_and_Microorganisms/Bacteria/Alphanumeric_Genus__Species/35319.aspx#culturemethod). The basal medium (initial pH 7.1) contained, per liter: Na_2_HPO_4_, 0.4 g; KH_2_PO_4_, 0.4 g; NH_4_Cl, 0.3 g; NaCl, 0.3 g; MgCl_2_, 0.1 g; CaCl_2_, 0.1 g; NaHCO_3_, 4.0 g; Na_2_S.9H_2_O, 0.2 g. The medium was supplemented with 0.2 mL/L of vitamin solution and 1 mL/L of acid and alkaline trace element solutions (each) [22]. 5 mg/L resazurin were added to the medium as a redox indicator. Inoculation was realized with 10% (v/v) of a pre-adapted culture grown on 1 g/L Sigmacell microcrystalline cellulose in 125 mL flasks with 50 mL working volume. Three cellulosic materials were used separately as sole carbon substrates: cotton pads (“Cotton”, Leader Price, Disques à Démaquiller—Simplement, Duo face, 100% cotton), Whatman qualitative filter paper, Grade 1 (“Whatman Paper”, 1001–125, 11 μm, 125 mm diameter) and paper handkerchief (“Tissue”, Lotus Classic, large handkerchiefs, made from pure cellulose fibers, pure virgin pulp). These substrates were cut in bands (~1.5 cm x 5 cm) and added to each flask to a final concentration of 2.5 g/L. For each cellulosic substrate separately, triplicate flasks were dedicated to physico-chemical monitoring of the degradation dynamics. For Tissue and Whatman paper separately, six additional replicate flasks by substrate were operated and sacrificed at 2 different time points for proteomic analyses during the incubation.

### Physico-chemical analyses of the incubation samples

At each sampling time, fresh samples (2 mL) were recovered from the 125 mL flasks and centrifuged at 10 000g for 10 min at 4°C. The obtained pellets and supernatants were stored separately at -80°C. Volatile fatty acid concentrations (including lactate and acetate) were measured using a DX 120 Ion Chromatograph (Dionex) with an IonPAc ICE-AS1 column. Ethanol concentrations were quantified by headspace gas chromatography—mass spectrometry (GC-MS) (Trace GC Ultra and DSQ II from Thermo with a TR-WAX column (30 m length, 0.25 mm intern diameter, 0.25 μm thick polyethylene glycol film).

### DNA extraction and quantification

DNA was extracted from the pellets with the PowerSoil DNA Isolation Kit (MO BIO Laboratories, Inc., Carlsbad, CA, USA) according to the manufacturer’s instructions and was quantified with the fluorescence-based Qubit dsDNA HS assay (Life Technologies). The obtained values were multiplied by a constant factor specific of each DNA extraction series to take into account the extraction yields. Based on *R*. *cellulolyticum* genome size, it was estimated that 1 ng of DNA corresponded to 227,703 genome copies.

### Physico-chemical characterization of the substrates

The total solids and volatile solids in each substrate were calculated from the moisture and ash content, determined by oven-drying, following instructions described in EN ISO 12879 and EN ISO 12880. Material elemental composition was analyzed using a Vario EL III (Elementar Analysensysteme GmbH, Hanau, Germany). Chemical oxygen demand was measured on solid substrates with the LCK 514 kit (Hach Lange) according to the manufacturer’s instructions.

To determine the crystallinity index, diffraction diagrams were monitored by recording X-ray diffraction diagrams every 10 min on a Bruker D8 Discover diffractometer. Cu Kα1 radiation (Cu Kα1 = 1.5405 Å), produced in a sealed tube at 40 kV and 40 mA, was selected and parallelized using a Gobël mirror parallel optics system and collimated to produce a 500 μm beam diameter. Crystallinity index was calculated based on [23], as follows:

$$Crystallinity\;index\;\left(\%\right)=\;\frac{\sum_{2\theta }{\left|U-A\right|}_{2\theta }}{\sum_{2\theta }{\left|C-A\right|}_{2\theta }\;}\;\times \;100$$

With A the value obtained for the amorphous standard, C the value obtained for crystalline standard and U the value obtained for the sample.

To determine the molar mass distribution of the cellulosic substrates, the latter were dissolved in N,N-dimethylacetamide (DMAc) and derivatized by tri-carbanilation using phenylisocyanate as reactant (reaction time 5 days at 40°C)[24]. The reaction was quenched with methanol and direct samples from the obtained solutions were analyzed after dilution in tetrahydrofurane (THF) in a size-exclusion chromatographic system (Viscotek TDA-302 apparatus) equipped with 3 Varian PLGel Mixed B columns (7.8×300) with a guard column. The coupled detection was UV at 260 nm, DRI, RALS/LALS/RI at 670 nm (laser 3 mW, 670 nm) and viscometer detector. DRI was used as concentration detector and UV as control. The chromatographic solvent was THF, injected concentrations were 1 mg/mL (injection volume 100 microliter), and the dn/dC of cellulose tricarbanilate in THF was taken at a predetermined value of 0.165. Data were treated by the OmniSec^™^ (4.5.6. version) program (Malvern Co.).

The lignocellulose sugar content of the samples was determined by the commercial facility of Celignis Analytical (http://www.celignis.com, Analysis Package P7, substrate hydrolysis followed by ion chromatography). Van Soest fractionation of the substrates was performed by the commercial facility of INRA Transfert Environnement as in [25] (https://www6.montpellier.inra.fr/it-e).

### Proteomic analyses

#### Total proteome and exoproteome extraction and shotgun MS/MS analyses

After 46 h and 70 h of incubation, 3 flasks for each substrate were sacrificed at each time point and sampled for proteomic analyses. The total volume, except 2 mL used for chemical analyses, was supplemented with 1 mM Phenylmethylsulfonyl Fluoride to inhibit protease activity and centrifuged at 6 000g for 20 min. The collected supernatant was further centrifuged at 13 000g for 15 min. Pellets from the first centrifugation round and supernatants from the second one were frozen in liquid nitrogen immediately and stored at -80°C. Proteins from the pellets were extracted as described in [26] with minor modifications. Briefly, cell disruption was performed by bead-beating of the pellets resuspended in 1X PBS using 0.1 mm silica and glass beads, and by a subsequent ultrasonication step. Proteins were extracted using liquid phenol and precipitated with ammonium acetate in methanol. After several washing steps, the protein pellets were resuspended in buffer (7 M urea, 2 M thiourea, 4% (w/v) CHAPS) as described in [9] and stored at -80°C. Culture supernatants were filtered through 0.22 μm PVDF membrane filters and proteins were precipitated and resuspended as described above. Protein concentrations were measured using an Agilent 2100 Bioanalyzer with the High Sensitivity Protein 250 kit following the manufacturer’s instructions.

For each sample, 5μg of each whole proteome were classically purified by SDS-PAGE, digested by trypsin in gel and analyzed by LC-MS/MS on a LTQ-Orbitrap Discovery mass spectrometer (Thermo Fisher, USA). Peptide separation was realized with an Ultimate 3000 RSLCnano system (Dionex, Voisins le Bretonneux, France) using a long gradient and a C18 column (Pepmap100, 0.075 x 50 cm, 100 Å, 2 μm, Thermo) during 188 min to enhance the resolution and the sensitivity of peptide detection by mass spectrometry. A detailed protocol is provided S1 File.

#### Protein database search and label free quantification

The data processing pipeline was designed using the TOPPAS software [27], part of the OpenMS project [28]. X!Tandem (directly provided in the OpenMS archive) was used to perform database searches in batch mode. Peak lists were created by an OpenMS dedicated tool with an additional processing step. Indeed, a precursor recalculation was computed for each tandem spectrum to improve the number of matches between a spectrum and a peptide sequence. While proteins were digested with trypsin, the analysis program allowed for 2 missed trypsin cleavage sites. Cysteine carbamidomethylation was set as a fixed modification; methionine oxidation and protein N-terminal acetylation were set as variable modifications for all X!Tandem searches. The mass tolerances in MS and MS/MS were set to 10 ppm and 0.6 Da respectively. Data were searched against a target/decoy concatenated database to obtain a false discovery rate (FDR) value at the peptide level. All identifications were validated with a final peptide FDR of 5% and a calculated protein FDR of 1%.

For the relative quantification based on eXtracted Ion Chromatogram (XIC), peaks were detected in each sample using the « PeakPickerCentroid » algorithm (OpenMS software). Validated identification data were matched with detected peaks. Peaks which were assigned to the same peptide sequence in different samples were used as anchors for retention time alignment between those samples. A Protein Abundance Index (PAI) was calculated and defined as the average of XIC area values from the three most intense peptides identified for a given protein.

#### Statistical analyses of the label-free quantification proteome datasets

All statistical analyses were performed using the programming language R on log_10_-transformed and quantile normalized PAI. The substrate, incubation time and substrate-by-incubation time interaction effects were assessed by Tobit models with a censoring threshold equal to the minimum non-zero value for a given protein. With y*_ijkl_ the normalized log_10_ PAI of protein *l*, for the substrate *S*_*i*_, incubation time *T*_*j*_ and replicate *k*, the model used for each protein is the following:

$$y_{ijkl}=\langle \begin{array}{c} \underset{i^{\prime }j^{\prime }k^{\prime }:\;y_{i\prime j\prime k\prime l}^{*}>0}{\text{min}}\left(y_{i\prime j\prime k\prime l}^{*}\right)\text{ if }y_{ijkl}^{*}=0\text{ and }\exists k^{\prime }:y_{ijk^{\prime }l}^{*}\neq 0\\ \;y_{ijkl}^{*}\text{ otherwise} \end{array}$$

$$\text{with }y_{ijkl}^{*}=\mu_{l}+\alpha_{il}S_{i}+\beta_{jl}T_{j}+\gamma_{ijl}{\left(S,T\right)}_{ij}+\varepsilon_{ijkl}$$

$$\text{and }\varepsilon_{ijkl}\sim N\left(0,\sigma_{l}^{2}\right)$$

Tobit regressions were computed using the Markov chain Monte Carlo algorithm from the R package MCMCpack [29]. Runs that did not pass the Heidelberger and Welch's convergence diagnostic [30, 31] were discarded. Substrate (*α*_*il*_) and interaction (*γ*_*ijl*_) effect coefficients as well as p-values were determined from the posterior distributions. P-values were adjusted for multiple comparisons using Benjamini and Hochberg’s false discovery rate [32]. The significance threshold used corresponds to a false discovery rate of 1%.

For the supernatants, the proteins with at least one statistically significant effect were filtered to select only proteins likely to be released in the extracellular milieu, cellulosomal proteins and secreted CAZYmes (see next section). The other proteins from the supernatants (cytoplasm/cell wall) were considered as originating from cell lysis.

### *In silico* predictions of protein sub-cellular localization

Prediction of sub-cellular localization was obtained from LocateP database (www.cmbi.ru.nl/locatep-db/cgi-bin/locatepdb.py) and by analyzing *R*. *cellulolyticum* protein sequences with the SurfG+ program 1.02 [33] with default parameter values with a local Galaxy instance (migale.jouy.inra.fr/galaxy/). The protein sequences were moreover analyzed with the last available version of specific tools: SignalP 4.1 server (www.cbs.dtu.dk/services/SignalP/), LipoP 1.0 server (www.cbs.dtu.dk/services/LipoP/), SecretomeP 2.0 server (www.cbs.dtu.dk/services/SecretomeP/), TMHMM server v. 2.0 (www.cbs.dtu.dk/services/TMHMM/), selecting the “Gram-positive” option whenever available. The results were manually examined and confronted to the knowledge on *R*. *cellulolyticum* proteins, in particular on cellulosomal proteins and cellulases [16, 21]. For ABC transporters, the subfamily name was retrieved from the Archaeal and Bacterial ABC Systems database (ABCdb database www-abcdb.biotoul.fr/) and for peptidases, the Peptidase database identity (MEROPS ID) was indicated (merops.sanger.ac.uk/).

The mass spectrometry proteomic data are available via ProteomeXchange, identifier PXD001051 and DOI 10.6019/PXD001051.

## Results and Discussion

### Growth and fermentation patterns

**Fig 1:**
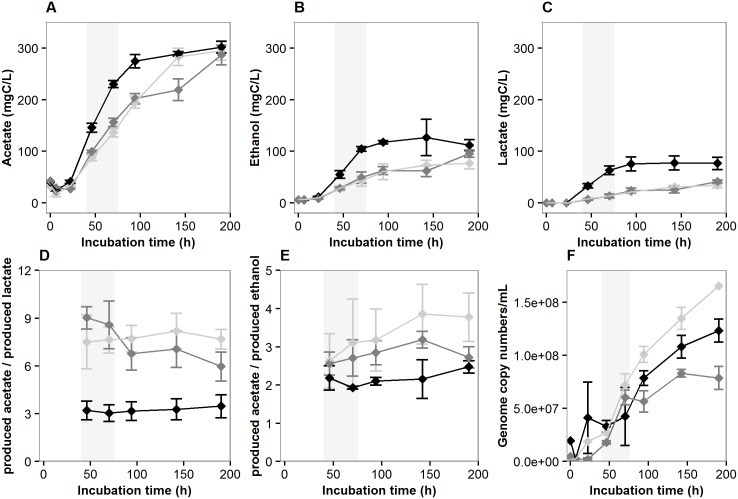
Growth and fermentation dynamics of *R*. *cellulolyticum* on Tissue (black symbols), Whatman Paper (grey symbols) and Cotton (light grey symbols). Acetate (A), ethanol (B) and lactate (C) are the three most abundant fermentation products and their concentration ratios are shown in (D-E). Genome copy numbers estimated from the amount of total extracted DNA are shown in (F). Error bars indicate standard deviations calculated from triplicate samples, except in F (duplicate samples). Light grey areas indicate the time points selected for subsequent proteomic analyses.

Fermentation dynamics of the three studied materials by *R*. *cellulolyticum* was characterized by incubating them separately in anaerobic, mesophilic conditions. For all incubations and as was expected [18, 20], acetate was the major end-product, followed by ethanol and lactate (Fig 1A–1C). Degradation occurred at a faster rate for Tissue than for both other substrates during the 94 first hours of incubation, as shown by the faster accumulation of acetate, ethanol and lactate in Tissue incubations over this time period (Fig 1A–1C, S1 Fig, panel A for the total dissolved organic carbon). A distinct metabolic profile was moreover observed for Tissue incubations, with lower acetate-to-lactate concentration ratios (Fig 1D) as well as lower acetate-to-ethanol concentration ratios compared to both other substrates (Fig 1E). This specific metabolic profile likely results from the higher sugar influx in the cells and the faster pH decrease in the milieu over time, from pH 7.1 to pH ~6.3 (S1 Fig, panel B). Indeed, it has previously been shown for *R*. *cellulolyticum* that the carbon flux partition between acetate, lactate and ethanol is greatly influenced by pH and entering carbon flows [20, 34, 35].

At the end of the 190 hours of incubation, the substrates were only partly degraded, as shown by the degradation yields estimated from carbon mass distributions (S1 File, S2 Fig and S1 Table), but significantly altered, disrupting easily during sample handling. Such a partial degradation is expected in batch microcosms where suboptimal growth conditions emerge over time, typically with the accumulation of H_2_ (values at the final time point shown in S1 Fig, panel C) or other fermentation products. Independently, colonization of the cellulosic substrates by the bacterial cells was qualitatively followed in microplate incubations over the incubation period, by wet mount microscopy and scanning electron microscopy (S1 File and S3 Fig). The observations were in good agreement with the degradation dynamics, since colonization occurred the fastest for Tissue and the slowest for Cotton. Scanning electron microscopy provided insight into the cell aggregate structure and suggested the absence of thick biofilm (S1 File and S3 Fig).

### Substrate characterization

To identify substrate’s properties likely to explain the differences observed during their fermentation by *R*. *cellulolyticum*, the three cellulosic substrates were characterized in details (Table 1). Measurement values of total solids, volatile solids, carbon, nitrogen and organic contents (Table 1) were each highly similar among the three substrates and consistent with cellulose being their major component. Moreover, the dominant chemical functional groups were the same in all substrates and typical of cellulosic materials since primary and secondary alcohols and glycosidic bonds were identified by Fourier transform infrared spectroscopy (FTIR, S1 File, S4 Fig).

**Table 1:** Detailed characteristics of Tissue, Whatman Paper and Cotton.

	Tissue	W. Paper	Cotton
**Total Solids/Volatil Solids (%/%)**	94.9/94.2	96.0/95.8	96.5/96.4
**Carbon/Nitrogen (%/%)**	41.9/0.07	43.1/0.03	41.1/0.05
**Chemical Oxygen Demand (g/g)**	1.06	1.14	1.11
**Crystallinity Index (%)**	50	94	74
Degree of Polymerization*	~970	~1300	~2730
**Total sugar content (% Dry Matter)**	**97.23**	**99.22**	**99.47**
**Hexoses (% Dry Matter)**	**83.35**	**98.67**	**98.94**
Glucose	81.54	98.58	98.69
Galactose	0.14	0.03	0.14
Mannose	1.66	0.05	0.07
Rhamnose	0.02	0.01	0.03
**Pentoses (% Dry Matter)**	**13.87**	**0.55**	**0.54**
Xylose	13.72	0.51	0.45
Arabinose	0.15	0.04	0.09
**Van Soest fractionation**			
Neutral Detergent Soluble fraction (%)	0.05	0.01	5.3
Acid Detergent Soluble fraction (%)	14.89	4.01	43.6
Sulfuric Acid Soluble fraction (%)	85.06	84.84	49.8
Insoluble Volatile Solids fraction (%)	0	11.13	1.2

W. Paper: Whatman Paper.

*The DP values correspond to the MW values of the peak of individual (i.e. non-aggregated) cellulose chains from the molar mass distribution plots (S5 Fig) divided by the mass of the tricarbanilated anhydroglucose unit (519 Da) (see Materials and Methods).

Important differences between the substrates concerned the crystallinity index (CI) (Table 1), the molar mass distributions (Table 1, S1 File and S5 Fig) and the hemicellulose content (Table 1). CI is commonly measured to estimate the amount of crystalline regions in cellulose, less easily degradable compared to amorphous regions. Based on the calculated CI, cellulose was the most amorphous in Tissue and the most crystalline in Whatman Paper (Table 1). The average degree of polymerization (DP) (Table 1, S1 File and S5 Fig) was the lowest for Tissue and the highest for Cotton. Both Whatman Paper and Cotton were composed almost exclusively of glucose (98.58% and 98.69% respectively, Table 1), whereas Tissue contained a significant proportion (13.87%) of pentoses (mainly xylose, 13.72%) in addition to the dominant glucose (81.54%) (Table 1). The composition data were consistent with the molecular weight distribution analyses (S1 File and S5 Fig).

Material characterization highlighted differences among the substrates both in terms of composition and structure. Based on these characteristics, the faster bioconversion observed for Tissue compared to both other substrates could arise from its low CI and its low average DP. Moreover, the presence of hemicelluloses in Tissue is likely to increase its enzyme accessibility and/or its hydrophilicity at the supramolecular level since networks of hemicelluloses and cellulose are less ordered and crystalline than networks of pure cellulose, which is thus likely to favor a faster degradation [36].

By contrast, Van Soest fractionation indicated that Whatman Paper is overall the substrate the less readily solubilized by chemical solutions (total of 4.02% solubilization by the first two detergents), followed by Tissue (total of 14.94% solubilization by the first two detergents) and Cotton (total of 48.9% solubilization by the first two detergents) (Table 1). These results highlight that great differences exist between chemical and biological reactivity for the studied cellulosic substrates.

### Comparative proteome-wide label-free quantification

To investigate which biological functions could predominantly be influenced by the substrate during fermentation, a sensitive shotgun proteomic approach based on XIC was implemented and served as basis for comparative quantitative analyses. Two substrates were selected for this approach, Tissue due to its specific metabolic profile and Whatman Paper as a reference since cellulose colonization by *R*. *cellulolyticum* has previously been studied on this substrate [37, 38]. Two time points were selected to be able to discriminate between the effect of the sole substrate and the effect of time and substrate interaction. Illustrative examples of such effects are provided in S6 Fig. Proteins were extracted from the pellets and supernatants separately at incubation times 46h and 70h (S7 Fig, panel A) and analyzed by LC-MS/MS. At the selected time points, fermentation products were just starting to significantly accumulate (Fig 1A–1C) and the biomass was actively growing (Fig 1F), limiting the possible effects of inhibitors accumulating in batch microcosms.

A total of 1194 proteins were quantified by the XIC approach (S1 Dataset). A good reproducibility was obtained (S7 Fig, panel B) and the identified functions were consistent with cellulose fermentation (S8 Fig, S2 Table). Comparative statistical analyses were conducted for each protein by adjusting models accounting for the influence of substrate, of time and of the interaction of substrate and time. In total, 151 proteins showing significantly different levels (Q-value < = 0.01) were identified and validated (S1 Dataset), including 132 with an effect in the pellets exclusively, 16 with an effect in the supernatants exclusively and 3 with an effect in both. They besides included 20 cellulosomal components (providing information about substrate hydrolysis mechanisms), 8 enzymes from the central carbon catabolism (providing information about the response to intracellular carbon fluxes) and 44 extracytoplasmic proteins (providing information about substrate transport and other specific functions).

#### Carbohydrate-active enzymes (CAZymes) and cellulosomal proteins

Considering their essential role in substrate deconstruction and catabolism, proteins related to cellulolysis and CAZymes were specifically examined. *R*. *cellulolyticum* cellulosomes include structural subunits and numerous different catalytic subunits [16, 17, 20] encoded by 65 distinct genes [21]. Their average protein composition is highly modulated according to the nature of the carbohydrate growth substrate [16, 21]. *R*. *cellulolyticum* genome encodes up to 149 CAZymes according to [21] and only a subset of them are extracellular components involved in lignocellulose deconstruction since CAZymes participate to a variety of other biological processes. For the present study, a total of 153 proteins from *R*. *cellulolyticum* were considered (S1 Dataset) corresponding to its CAZymes and to its known non-CAZyme cellulosomal structural subunits. Among these 153 proteins, 103 were quantified in at least one sample and 28 showed significantly different levels when comparing growth on Tissue and Whatman Paper (Tables 2–4). The present approach appears as complementary to specific proteomic approaches targeting cellulosomal components (such as in [16]) since the sensitivity is comparable and additional functions can also be detected. Indeed, out of the 52 cellulosomal components and CAZymes detected in [16], 47 were quantified in the present study and, for instance, 7 additional proteins with a dockerin-module (cellulosomal components), not detected in [16], were quantified here.

**Table 2:** Cellulosomal endoglucanases with significantly different levels when comparing Tissue and Whatman Paper incubations.

		Pellet		Supernatant				
Gene ID	Protein/Gene name	Substrate^a)^	Interaction^b)^	Substrate^a)^	Interaction^b)^	Modular structure^c)^	Localization^d)^	Protein function or name^e)^
Ccel_1648	β-glucanase R, Cel9R	**-0.24**			**+0.29**	S-GH9-CBM3-UNK-CBM3-DOC1	**cellulosome**	***Endoglucanase Cel9R***
Ccel_0732*	Cel9E	**-0.26**				S-UNK-CBM4-UNK-GH9-UNK-DOC1	**cellulosome**	**Endoglucanase/cellobiohydrolase Cel9E**
Ccel_0734*	Cel9H	**-0.18**				S-UNK-GH9-UNK-CBM3-DOC1	**cellulosome**	***Endoglucanase Cel9H***
Ccel_1249	β-glucanase T, Cel9T	**-0.25**				S-GH9-CBM3-DOC1	**cellulosome**	***Endoglucanase Cel9T***
Ccel_0735*	Cel9J	**-0.42**	**-0.38**			S-GH9-CBM3-DOC1	**cellulosome**	***Endoglucanase Cel9J***
Ccel_0740*	Cel5N	**-0.28**	**-0.25**			S-GH5-DOC1	**cellulosome**	Endoglucanase Cel5N
Ccel_0753	Cel9P, P90	**-0.22**	**-0.28**			S-GH9-CBM3-UNK-DOC1	**cellulosome**	***Endoglucanase Cel9P***
Ccel_0755	cellulase U / cellulase S, Cel9U		**-0.13**			S-UNK-GH9-DOC1	**cellulosome**	***Endoglucanase Cel9U***
Ccel_2392	Cel9V		**-0.21**			S-UNK-CBM4-UNK-GH9-DOC1	**cellulosome**	***Endoglucanase/cellobiohydrolase Cel9V***
Ccel_1099	celCCA, Cel5A, Cca			**-3.63**	**-3.63**	S-GH5-DOC1	**cellulosome**	**Endoglucanase/endoxylanase Cel5A**
Ccel_2337	CMCase, P66	**+0.28**				S-GH5-DOC1	**cellulosome**	Endoglucanase
Ccel_0429	P99			**+0.40**		S-GH44-DOC1-UNK-CBM44	**cellulosome**	Endoglucanase Cel44O (PKD domain containing protein)

The proteins are listed according to the observed effects and to their function. The statistical models take into account the replicates and their variability.

* in the first left column (“Gene ID”) indicate genes encoded in the “*cip-cel*” gene cluster, which codes for 12 key cellulosomal components.

^a)^ The log10 fold change values are indicated for the proteins with statistically significant substrate effects (Q-value < = 0.01). Tissue incubations are used as a reference (positive values when the protein levels are higher in the Tissue incubations).

^b)^ The log10 fold change values are indicated for the proteins with statistically significant substrate-by-time interaction effects (Q-value < = 0.01). Tissue incubations are used as a reference (positive values when the protein levels increase faster or decrease slower in the Tissue incubations).

^c)^ According to [16, 21] and the present study: S: signal sequence; GH: family of glycoside hydrolase; PL: family of pectate lyase; CE: family of carbohydrate esterase; GT: family of glycosyl transferase; CBM: family of carbohydrate-binding module; DOC1: dockerin type 1 module; COH: cohesin type I module, LNK: linker sequence; SLH: surface-layer homology sequence; COG: clusters of orthologous groups; UNK: unknown function module or sequence; TSP_C: thrombospondin C-terminal region; fn3: fibronectin type III domain.

^d)^ Known (in bold) or predicted localization.

^e)^ Predicted or characterized (in bold) activities or protein names according to [16] and to UniprotKB database.

**Table 3:** Other cellulosomal proteins with significantly different levels when comparing Tissue and Whatman Paper incubations.

		Pellet		Supernatant				
Gene ID	Protein/Gene name	Substrate	Interaction	Substrate	Interaction	Modular structure	Localization	Protein function or name
Ccel_0931	P41a, xyn10A	**-0.31**				S-GH10-DOC1	**cellulosome**	Xylanase Xyn10A
Ccel_2162	P42	**-0.27**				S-DOC1-CE2	**cellulosome**	Acetyl-xylan esterase
Ccel_1655		**-0.22**				S-DOC1-UNK	**cellulosome**	Unknown (cellulosome protein dockerin type I)
Ccel_1060		**-4.09**	**-3.78**			S-COG2755 / COG2845-DOC1	**cellulosome**	SGNH-hydrolase
Ccel_2243		**-0.23**	**-0.22**			S-PL1-UNK-DOC1-UNK	**cellulosome**	Pectate lyase
Ccel_0379	P76		**-0.20**			S-GH5-LNK-CBM32-DOC1	**cellulosome**	Mannanase
Ccel_1597	P50		**-0.15**			S-GH27-UNK-DOC1	**cellulosome**	α-Galactosidase
Ccel_1543				**+0.60**		S-TSP_C–(fn3)4-CBM	**cellulosome**	Cellulosome anchoring protein cohesin region

The column titles are identical to those from Table 2.

**Table 4:** Non-cellulosomal CAZymes with at least one statistically significant effect when comparing incubations on Tissue and Whatman Paper.

		Pellet		Supernatant				
Gene ID	Protein/Gene name	Substrate	Interaction	Substrate	Interaction	Modular structure	Localization	Protein function or name
Ccel_0428	Cel5I			**-3.85**	**+3.83**	S-GH5-CBM17-CBM28-(SLH)3	cell wall	Endoglucanase Cel5I
Ccel_2417				**+3.86**	**+3.86**	GT39	cell wall	Glycosyl transferase family 39
Ccel_0881				**-0.60**		S-CBM16-UNK	secreted	Unknown (Carbohydrate-binding, CenC-like protein)
Ccel_1036				**-0.27**		GH51-UNK	secreted	α-Arabinofuranosidase
Ccel_2893				**-3.64**	**+3.64**	S-GH18-UNK	secreted	β-Glycosidase
Ccel_1139		**+0.30**				UNK-GH3-UNK	intracellular	β-Glucosidase
Ccel_0203			**+0.31**			GH3-UNK	intracellular	β-Xylosidase
Ccel_3438			**+0.34**			GH43-UNK	intracellular	β-Xylosidase/a-arabinofuranosidase

The column titles are identical to those from Table 2.

As expected, the 28 proteins with significantly different levels were dominated by cellulosomal subunits (Tables 2 and 3, total of 20 proteins). The levels of an important proportion of the 65 cellulosomal proteins were thus significantly influenced although both cellulosic substrates are rather similar, highlighting the sensitivity of cellulosome composition to subtle substrate differences. Among the 20 cellulosomal proteins with significant effects, 17 had lower levels or levels that decreased faster when comparing growth on Tissue and on Whatman Paper (Tables 2 and 3, at least one negative log fold change), encompassing 6 glycoside hydrolase (GH) families and 2 other CAzyme families (Tables 2 and 3). This observation is at first sight unexpected: minor sugar components being more abundant in Tissue compared to Whatman, especially hemicelluloses (Table 1), it could have been anticipated that a variety of cellusome enzymes could have higher levels in the Tissue incubations. In the model proposed by [21], core cellulosomal genes are activated, or not repressed, when intracellular levels of glycolytic intermediates are low (carbon catabolite repression) [21]. Since less carbon flowed through glycolysis during growth on Whatman paper compared to Tissue, results obtained here reflect that hydrolysis of the most recalcitrant substrate requires more diverse cellulosomal enzymes during a longer time.

Hemicelluloses from Tissue were very likely at least partly fermented during the incubations since 3 intracellular proteins involved in xylose or xylose oligomer catabolism (encoded by Ccel_0203, Ccel_3438, Table 4, and Ccel_3429) had higher levels in Tissue incubations, suggesting a higher xylodextrin intracellular influx therein. It is however unclear whether the higher abundance of xylose in Tissue specifically induced the higher expression of certain xylanases. Indeed, cellulosomal proteins encoded by the “*xyl-doc”* cluster (14 genes more specifically oriented towards hemicellulolysis [16]) did not show any significant differences, although 8 of them were quantified in the whole dataset (S1 Dataset). The cellulosomal endoglucanase Ccel_0429, presenting higher levels in Tissue incubations, could have participated to hemicellulose deconstruction since it exerts xyloglucan depolymerization as a secondary activity [39]; however its regulation mechanisms are unknown. Finally, the other cellulosomal subunits with a known xylanase activity and statistically significant differences had lower levels in Tissue incubations (Ccel_0755 in Table 2, Ccel_0931 in Table 3).

The lack of specific activation of the “*xyl-doc”* cluster has previously been reported [16, 21] during growth of *R*. *cellulolyticum* on oat spelt xylan, as well as its activation during growth on wheat straw [16] or corn stover [21]. Together with the present work, these observations suggest that natural lignocellulosic substrates, in which hemicellulose is associated to lignin within complex entangled structures, are more likely to induce the expression of the “*xyl-doc”* cluster than more simple or engineered materials where the growth substrates are more readily available.

The subset of cellulosomal proteins with significantly different levels was enriched in endoglucanases (12 out of 20 cellulosomal proteins with different levels, Table 2, compared to 17 endoglucanases in total among the 65 cellulosomal proteins annotated in *R*. *cellulolyticum* genome). Among these endoglucanases, 10 had significantly lower levels in the Tissue incubations, particularly enzymes from the GH5 and GH9 families (Table 2). This result is consistent with the presence of shorter cellulose chains in this Tissue as well as its lower CI. In the closely related *Ruminiclostridium thermocellum* [40] and *Ruminiclostridium clariflavum* [41], it has indeed been shown that the levels GH9 endoglucanases, which are also very common in their cellulosomes, are influenced by the substrate’s nature, with increased levels in the presence of crystalline cellulose.

Overall, CAZyme expression appears to be influenced here more by substrate structure than by its carbohydrate composition, although the present experiments do not provide a fully formal proof.

#### Central carbon metabolism

**Fig 2:**
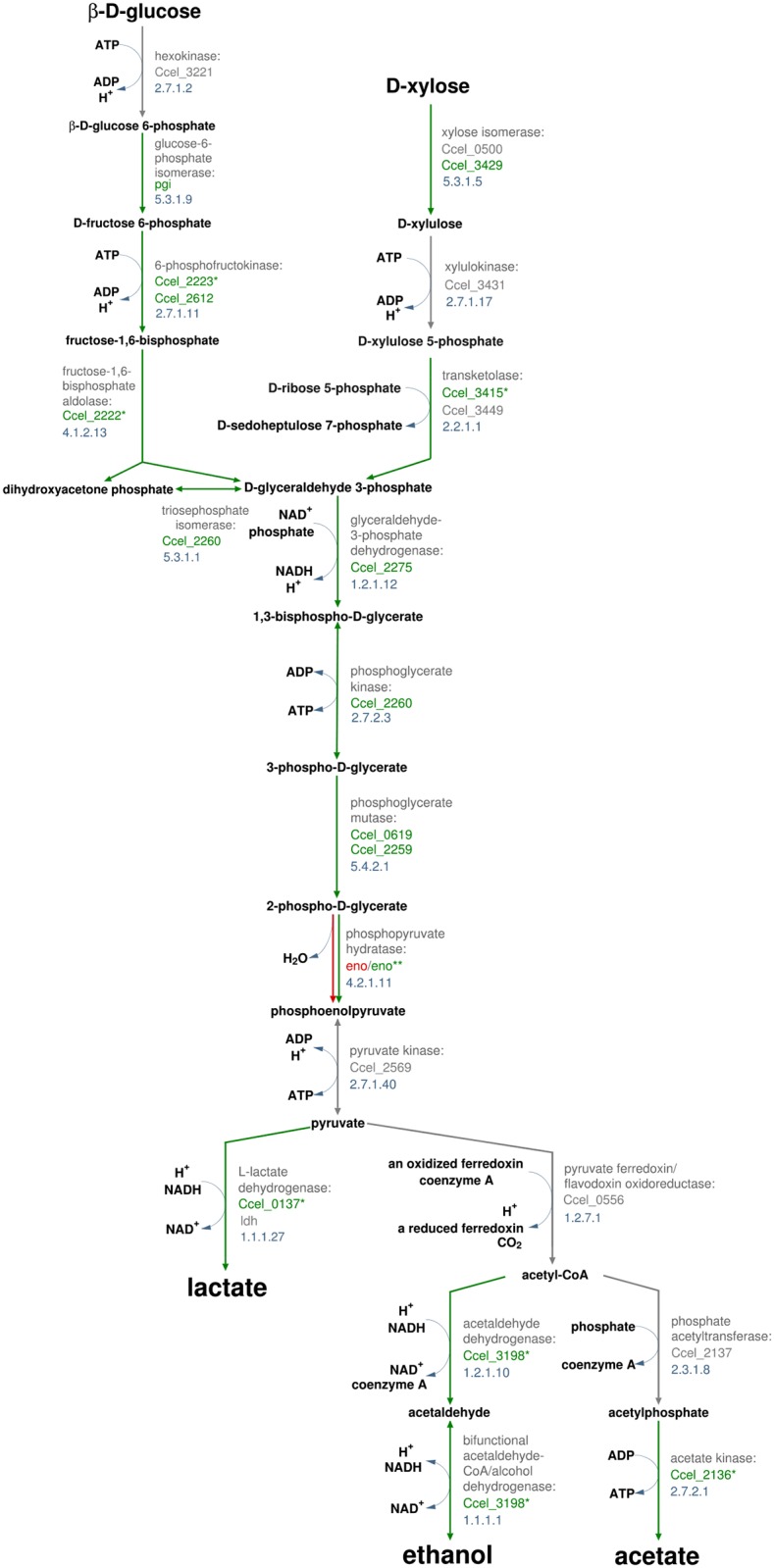
Proteins with significant effects when comparing growth on Tissue and Whatman Paper mapped over *R*. *cellulolyticum* glucose and xylose catabolic pathways. Statistically significant substrate and substrate-by-time interaction effects were considered. The green color indicates positive effects while the red color indicates negative effects. Positive effects correspond to quantified protein levels higher in Tissue incubations than in Whatman Paper incubations (substrate effect) and to quantified protein levels increasing more or decreasing less in Tissue incubations than in Whatman Paper incubations (substrate-by-time interaction effect). The statistical models (see Materials and Methods) take into account the replicates and their variability. Protein names and EC numbers are indicated in grey. * indicate effects not significant when adjusting for multiple comparisons (Q-values > 0.01) but still supporting the overall trend (p-values < = 0.05). ** indicate a significant negative substrate effect (q-value ≤ 0.01) and a positive interaction effect (q-value > 0.01 but p-value ≤ 0.05). Pathways were adapted from the Biocyc website (http://biocyc.org/).

To determine whether the level of sugar influx affected the expression of enzymes from the central carbon metabolism, enzymes from the glucose and xylose catabolic pathways of *R*. *cellulolyticum* (Fig 2 and S1 Dataset) were specifically examined. Among them, 23 were successfully quantified in at least one of the pellet samples and statistical models could be adjusted for 18 of them. Finally, 8 proteins showed statistically significant effects: the glucose-6-phosphate isomerase (product from Ccel_1445, pgi), the ATP-dependent 6-phosphofructokinase (Ccel_2612, pfkA), the xylose isomerase (Ccel_3429, xylA), the glyceraldehyde-3-phosphate dehydrogenase (Ccel_2275), the phosphoglycerate kinase (Ccel_2260), the phosphoglycerate mutase (Ccel_0619), the 2,3-bisphosphoglycerate-independent phosphoglycerate mutase (Ccel_2259, gpmI) and the phosphopyruvate hydratase, also known as enolase (Ccel_2254, eno). All 8 of them are enzymes from upstream of the pyruvate node and they are distributed over the Embden-Meyerhof-Parnas (EMP) and xylose-utilization pathways (Fig 2). Except for the enolase, they all showed only positive effects, indicating higher protein levels and/or lower protein level decreases in Tissue incubations compared to Whatman Paper incubations.

This result shows that the higher sugar influx during Tissue fermentation leads to an overall enhanced expression of enzymes from the carbon catabolic pathways, which is shown for the first time here for *R*. *cellulolyticum* and contrasts to a previous report on the closely related *Ruminiclostridium termitidis* [42].

#### Other extracytoplasmic proteins

The nature of the cellulosic substrate most certainly directly or indirectly influences the expression levels of numerous extracytoplasmic proteins since the latter are involved in a variety of biological processes in bacteria, including substrate colonization, substrate uptake, or cell-cell interactions, which are all relevant for the present study. Predicting the subcellular localization of proteins is a complex issue [43]. To identify extracytoplasmic proteins in the present dataset, the 115 proteins showing at least one statistically significant effect when comparing growth on Tissue and Whatman Paper (other than those already described in the result section on CAZymes and cellulosomal proteins) were specifically analyzed *in silico*. Among the 115 manually examined proteins, 44 had a predicted extracytoplasmic localization and the detailed results are shown in S3 Table. For 36 of these 44 proteins, the prediction can be considered as very robust since at least both SurfG+ and LocateP indicate an extracytoplasmic localization (with however sometimes distinct predicted subcellular localizations).

Noticeably, the 44 selected proteins include 8 components of ATP-binding Cassette (ABC) transporters (Table 5) and 4 of them, encoded by Ccel_1987, Ccel_2997, Ccel_0998 and Ccel_1133, are likely involved in sugar transport based on blastp analyses against the manually curated part of ABCdb (www-abcdb.biotoul.fr, CleanDb). They indeed show significant similarity (e-value < 10^−5^) with the sequence of several proteins annotated as sugar binding proteins and with at least one experimentally well-characterized sugar-transporter component such as the D-xylose transporter subunit encoded by the xylF gene from *Escherichia coli* (Ccel_1987) or the multiple sugar-binding ABC transporter, sugar-binding protein precursor MsmE encoded by msmE gene from *Streptococcus mutans* (Ccel_2997, Ccel_0998, Ccel_1133). The trends regarding their expression are contrasted, since higher or lower levels are observed in Tissue incubations according to the protein (Table 5). These results show the modulation of the sugar ABC transporter profile according to the nature of the cellulosic substrate and consistently, genes Ccel_1987, Ccel_0998 and Ccel_1133 belong to genomic regions regulated by Two-Components Systems responding to the availability of specific extracellular soluble sugars, as described in [21].

**Table 5:** ABC transporter proteins with significantly different levels when comparing Tissue and Whatman Paper incubations.

		Pellets		Supernatants			
Gene ID	Protein name	Sub. ^a)^	Inter. ^b)^	Sub. ^a)^	Inter. ^b)^	SurfG+^c)^	LocateP^d)^
Ccel_1987	Putative solute-binding component of ABC transporter *(S_1ab)*	+0.37				PSE	Lipid anch.
Ccel_1133	Extracellular solute-binding protein family 1 *(S_5ab)*	+0.55				PSE	Lipid anch.
Ccel_1768	Extracellular solute-binding protein family 5 *(S_2a)*		+0.27			PSE	Lipid anch.
Ccel_2997	Extracellular solute-binding protein family 1 *(S_5ab)*	-7.65		NA	NA	PSE	N-ter anch.
Ccel_0967	Transport permease protein *(M_7a)*	-0.15		NA	NA	MB	Memb.
Ccel_0998	Extracellular solute-binding protein family 1 *(S_5ab)*	-3.66	+3.66	NA	NA	PSE	Lipid anch.
Ccel_1156	Periplasmic solute binding protein *(S_8b)*	-3.68	+3.68	NA	NA	PSE	Lipid anch.

The proteins are listed according to the observed effects and to the subcellular localization predicted by SurfG+.

^a)^ The log10 fold change values are indicated for proteins with statistically significant substrate effects (“Sub., Q-value < = 0.01). Tissue incubations are used as a reference (positive values when the protein levels are higher in the Tissue incubations). The statistical models take into account the replicates and their variability.

^b)^ The log10 fold change values are indicated for proteins with statistically significant substrate-by-time interaction effects (“Inter.”, Q-value < = 0.01). Tissue incubations are used as a reference (positive values when the protein levels increase faster or decrease slower in the Tissue incubations). The statistical models take into account the replicates and their variability.

^c)^ Subcellular localization predicted by SurfG+. PSE: potentially surface exposed; MB: membrane.

^d)^ Subcellular localization from LocateP database. Lipid anch.: Lipid anchored; Memb: Multi-transmembrane;. N-ter anch.: N-terminally anchored (No cleavage site).

Interestingly, a Fibronectin type III domain protein (encoded by Ccel_0648) predicted to be released in the extracellular milieu by both SurfG+ and LocateP was associated to significant negative effects in the pellets, indicating higher levels in the Whatman paper incubations (S3 Table). A Fibronectin type III-like repeat from the *Ruminiclostridium thermocellum* cellobiohydrolase CbhA was previously shown to promote hydrolysis of cellulose by modifying its surface [44]. If the protein encoded by Ccel_0648 participates to a similar function, its higher concentration levels in Whatman Paper incubations could favor the hydrolysis of this more recalcitrant substrate compared to Tissue.

Other extracytoplasmic proteins with significantly different levels could be interesting, such as those containing SLH domains; however, a fine interpretation is overall hindered by the currently limited knowledge on non-cellulolytic extracytoplasmic proteins in *R*. *cellulolyticum*. For instance, the ABC transporter specificities are poorly described for this species. Strikingly, among the 16 proteins of unknown function present in the dataset of the 151 significant proteins, 13 correspond to predicted extracytoplasmic proteins (S3 Table). Better characterizing transporters and other extracytoplasmic proteins from *R*. *cellulolyticum* thus appears of great importance to better understand its physiology during cellulose degradation and to be able to implement global approaches such as systems metabolic engineering.

## Conclusions

Fermentation by model cellulolytic bacteria of engineered materials has been little studied so far. In this study, fermentation by *R*. *cellulolyticum* of three cellulosic substrates containing no lignin, paper handkerchief, cotton discs and Whatman filter paper was considered. Paper handkerchief was fermented the fastest and 151 proteins had significantly different levels when comparing paper handkerchief and Whatman filter paper incubations, including 8 enzymes from the central carbon metabolic pathways and 44 distinct extracytoplasmic proteins. They moreover comprised 20 out of the 65 cellulosomal components and 4 non-cellulosomal extracytoplasmic CAZymes potentially involved in cellulolysis, highlighting the sensitivity of the cellulolysis machinery to subtle differences in substrate properties. In particular, ten cellulosomal endoglucanases, mainly from GH5 and GH9 families, had lower levels during fermentation of paper handkerchief when comparing with fermentation of Whatman paper. This observation hypothetically results from the lower crystallinity rate and degree of polymerization of cellulose in paper handkerchief. Paper handkerchief exhibited higher hemicellulose content and the enhanced level of intracellular xylose isomerase suggested that the hemicellulose was at least partly metabolized. However, regarding hemicellulose hydrolysis, none of the known extracytoplasmic enzymes with xylanolysis as primary activity had significantly higher levels in the Tissue incubations. It appears that natural lignocellulosic substrates, in which hemicellulose is associated to lignin within complex entangled structures, could be more likely to induce the expression of the “*xyl-doc”* cluster or other specialized xylanases than more simple or engineered materials where the growth substrates are more readily available. Similar to differences occurring among Tissue and Whatman paper incubations, there could be significant differences on protein levels among Whatman paper and Cotton incubations, especially regarding the cellulolysis machinery, since these substrates have different crystallinity index and degrees of polymerization. Addressing this question would require further proteomic analyses. The present study provides, to our knowledge, the first whole-proteome analysis on the model cellulolytic bacterium *R*. *cellulolyticum* and expands the knowledge on the proteome response of this bacterium to cellulosic substrates.

## Supporting Information

S1 Fig Data on fermentation of Tissue (black symbols), Whatman Paper (grey symbols) and Cotton (light grey symbols) by *R*. *cellulolyticum*. (A) Evolution over time of the total Dissolved Organic Content (DOC). (B) Evolution over time of pH. (C) Cumulated gas production at the final incubation time point. Error bars indicate standard deviations calculated from triplicate samples. Light grey areas in (A) and (B) indicate the time points selected for subsequent proteomic analyses. *R*. *cellulolyticum* was grown in 50 mL batch fermentation microcosms on 2.5 g/L cellulosic substrate.
(TIF)

S2 Fig Average carbon mass distributions in the microcosms at the initial and final incubation time points. The carbon masses are in mg. Sampled: carbon mass removed from the microcosms through sampling of the liquid phase—CO2 gas: carbon mass in CO_2_ in the headspace—DIC: inorganic carbon mass in the liquid phase (Dissolved Inorganic Carbon)–DOC: organic carbon mass in the liquid phase (Dissolved Organic Carbon)–Cellulosic material: estimated carbon mass in the substrate (contained either in Tissue, Whatman Paper or Cotton). The percent values next to each substrate name indicate the estimated average degradation yield (percentage of carbon from the substrate that was degraded). Details on the calculation method are available in S1 File.
(TIF)

S3 Fig Illustrative microscopy images of substrate colonization by *R*. *cellulolyticum* during growth in microplates on Tissue (left column), Whatman Paper (middle column) and Cotton (right column) at a final concentration of 5 g/l. Confocal Laser Scanning Microscopy images were acquired from Tissue (A-D), Whatman Paper (F-I) and Cotton (K-N) incubations, on wet mount samples stained with a cellular esterase activity marker (green) after removal of the planktonic cells. Scale bars are 200 μm. A total of 75 representative images were acquired. Scanning Electron Microscopy images were acquired from Tissue (E), Whatman Paper (J) and Cotton (O) incubations, on samples collected after 48 h of incubation. Scale bars are 3 μm. A total of 137 images of scanning electron microscopic were acquired. Details on the methods are available in S1 File.
(TIF)

S4 Fig Mid-infrared absorption spectra obtained for Tissue, Whatman Paper and Cotton. W. Paper: Whatman Paper. The 5 peaks annotated with arrows on the spectra are related to the presence of cellulose. Details on the method are available in S1 File.
(TIFF)

S5 Fig Molar mass distribution curves of the cellulose and hemicellulose chains from Tissue (black lines), Whatman Paper (grey lines) and Cotton (light grey lines). M stands for Molar Mass. Vertical colored lines indicate the positions of the M values corresponding to the peak of individual (i.e. non-aggregated) cellulose chains (see Table 1). Grey area A1: hemicellulose distribution peak observed for Tissue, originating from bleached wood pulp. Grey area A2: peaks corresponding to very high molecular weight polymers and, more likely, to cellulose chain aggregates. Details on the method are available in S1 File.
(TIFF)

S6 Fig Illustrative examples of statistically significant substrate and substrate-by-time interaction effects on the protein levels. The illustrative examples are selected from the dataset of the pellet proteins. On each dot plot, the values are shown for the incubation times 46h, 70h and for the blank. The shown values correspond to the log-transformed and normalized data. The red color corresponds to Tissue incubations and the green color to Whatman Paper incubations. The “+” and “-”signs indicate the sign of the considered effect (substrate or substrate-by-time interaction). From left to right and from top to bottom: Ccel_1139 encodes a β-Glucosidase (see also Table 4); Ccel_0734 encodes the endoglucanase Cel9H (see also Table 2); Ccel_0203 codes for a β-Xylosidase (see also Table 4); Ccel_2392 codes for the endoglucanase/cellobiohydrolase Cel9V (see also Table 2); Ccel_1570 encodes a putative uncharacterized protein; Ccel_0735 encodes the endoglucanase Cel9J (see also Table 2); Ccel_3392 encodes a putative uncharacterized protein; Ccel_0428 encodes the endoglucanase Cel5I (see also Table 4).
(TIF)

S7 Fig Quantification of total extracted proteins and principal component analyses of the proteomes based on the individual protein quantification data. A) Total amounts of proteins extracted from the Tissue (black) and Whatman Paper (grey) incubations, after 46h and 70h of incubation, from the pellets and supernatants respectively. B) Principal component analysis of the samples based on the label-free quantitative proteomic data (XIC approach).
(TIF)

S8 Fig Functional profiles for all proteins encoded in *R*. *cellulolyticum* genome and for the proteins showing significantly different levels in the presence of Tissue compared to Whatman Paper. A selection of 32 Gene Ontology (GO) terms is shown, corresponding to the categories with highest percentages of annotations and to the most enriched or depleted categories when comparing the dataset of proteins with significantly different levels (after removal of categories with less than 3 proteins with significantly different levels) and all genome-encoded proteins. The GO terms are shown from the most enriched to the most depleted, from top to bottom. *R*. *cellulolyticum* genome encodes 3290 proteins, of which 2081 have GO annotations in UniprotKB, corresponding to a total of 3674 GO annotations. 151 proteins showed significantly different levels, of which 116 have GO annotations in UniprotKB, corresponding to a total of 385 annotations. Numeric values and additional details are shown in S2 Table.
(TIF)

S1 Dataset Summary of the quantitative results obtained for each protein of *R*. *cellulolyticum*. (XLSX)

S1 Table Carbon mass distributions in the microcosms at the initial and final incubation time points. The carbon masses are in mg. The average values (± one standard deviation where relevant) are shown. Cellulosic material: carbon mass in the substrate (contained either in Tissue, Whatman Paper or Cotton)–DOC: organic carbon mass in the liquid phase (Dissolved Organic Carbon)–DIC: inorganic carbon mass in the liquid phase (Dissolved Inorganic Carbon)–CO2 gas: carbon mass in CO2 in the headspace—Sampled: carbon mass removed from the microcosms through sampling of the liquid phase—Total: total carbon mass in the microcosms at the initial incubation time point—Degradation yield: estimated percentage of degraded carbon from the substrate. The carbon mass in the cellulosic material at the final time point is calculated by considering that the total carbon mass is identical at time points 0h and 190h in the system. To calculate the carbon mass removed through sampling of the liquid phase, two options were considered: no substrate particles were sampled (option 1), substrate particles at a concentration of 2.6 g/L (corresponding to the initial concentration) were sampled (option 2). Consequently, two different values were obtained for the carbon mass in the cellulosic material at time point 190h. More details on the method are available in S1 File.
(PDF)

S2 Table Functional profiles for all proteins encoded in *R*. *cellulolyticum* genome and for proteins showing significantly different levels in the presence of Tissue compared to Whatman Paper. a) BP: Biological Process—CC: Cellular Component—MF: Molecular Function. b) For each GO term, percentage of the GO term annotations among the significant proteins. c) For each GO term, percentage of the GO term annotations among all proteins encoded in *R*. *cellulolyticum* genome. d) Ratios of both percentages. The GO terms are presented by decreasing ratio values. See legend from S7 Fig for additional details.
(XLSX)

S3 Table Extracytoplasmic proteins with at least one statistically significant effect when comparing incubations on Tissue and Whatman Paper. The proteins are listed according to the observed effects, to the subcellular localization predicted by SurfG+ and according to their function. a) The Gene IDs (Ccel_) are given according to UniprotKB database. b) The Protein names are given according to UniprotKB database. c)—d) Statistically significant effects for proteins quantified in Pellets and Supernatants respectively. Substrate effects (Q-value < = 0.01) are indicated with + for positive effects (quantified protein levels higher in Tissue incubations than in Whatman Paper incubations) and—for negative effects (quantified protein levels lower in Tissue incubations than in Whatman Paper incubations). Substrate-by-time interaction effects (Q-value < = 0.01) are indicated with + for positive effects (quantified protein levels in Tissue incubations increase more or decrease less than in Whatman Paper incubations) and—for negative effects (quantified protein levels in Tissue incubations increase less or decrease more than in Whatman Paper incubations).The statistical models take into account the replicates and their variability. e) Subcellular localization predicted by SurfG+. PSE: potentially surface exposed; EXT: extracellular milieu; CYTO: cytoplasm; MB: membrane. f) Subcellular localization from LocateP database. Lipid anch.: Lipid anchored; Released: Secretory (released) (with cleavage site); Memb: Multi-transmembrane;. N-ter anch.: N-terminally anchored (No cleavage site); Intracell.: Intracellular. g) SignalP predictions concerning the presence of a signal peptide. Y: yes; N: no. h) SecretomeP predictions concerning the secretion by a non-classical pathway (without signal peptide). Y: yes; N: no. i) LipoP predictions concerning lipoproteins and signal peptides. SpII: lipoprotein signal peptide (signal peptidase II); SpI: signal peptide (signal peptidase I); TMH: n-terminal transmembrane helix (this is generally not a very reliable prediction according to LipoP website); CYT: cytoplasmic (all others). j) TMHMM predictions: number of predicted transmembrane helixes. k) TMHMM predictions: possible presence of a signal peptide (as indicated on THMM server website, “predicted TM segments in the N-terminal region sometime turn out to be signal peptides”).
(XLSX)

S1 File Supplementary Materials and Methods and References. (PDF)
