# Supplementary figures and images for: Whole Proteome Analyses on Ruminiclostridium cellulolyticum Show a Modulation of the Cellulolysis Machinery in Response to Cellulosic Materials with Subtle Differences in Chemical and Structural Properties
Source: PLoS One. 2017 Jan 23;12(1):e0170524. doi: 10.1371/journal.pone.0170524 (PMC5256962; doi:10.1371/journal.pone.0170524)

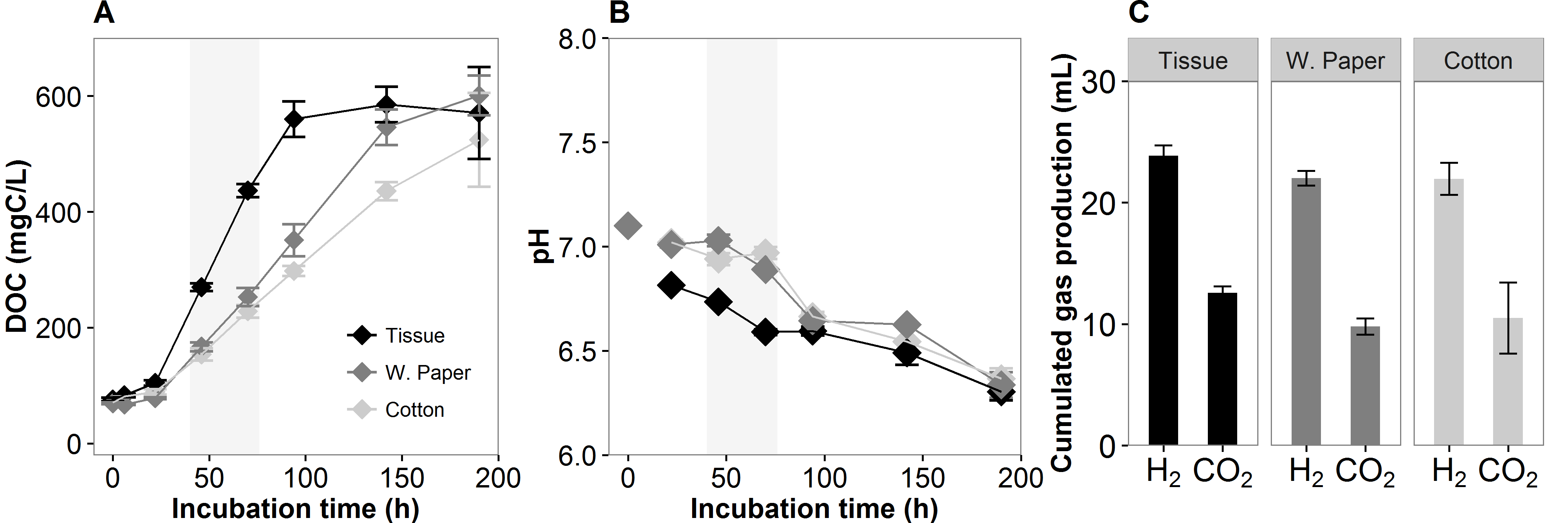

Supplement: S1 Fig — (A) Evolution over time of the total Dissolved Organic Content (DOC). (B) Evolution over time of pH. (C) Cumulated gas production at the final incubation time point. Error bars indicate standard deviations calculated from triplicate samples. Light grey areas in (A) and (B) indicate the time points selected for subsequent proteomic analyses. R. cellulolyticum was grown in 50 mL batch fermentation microcosms on 2.5 g/L cellulosic substrate. (TIF) [file pone.0170524.s001.tif]

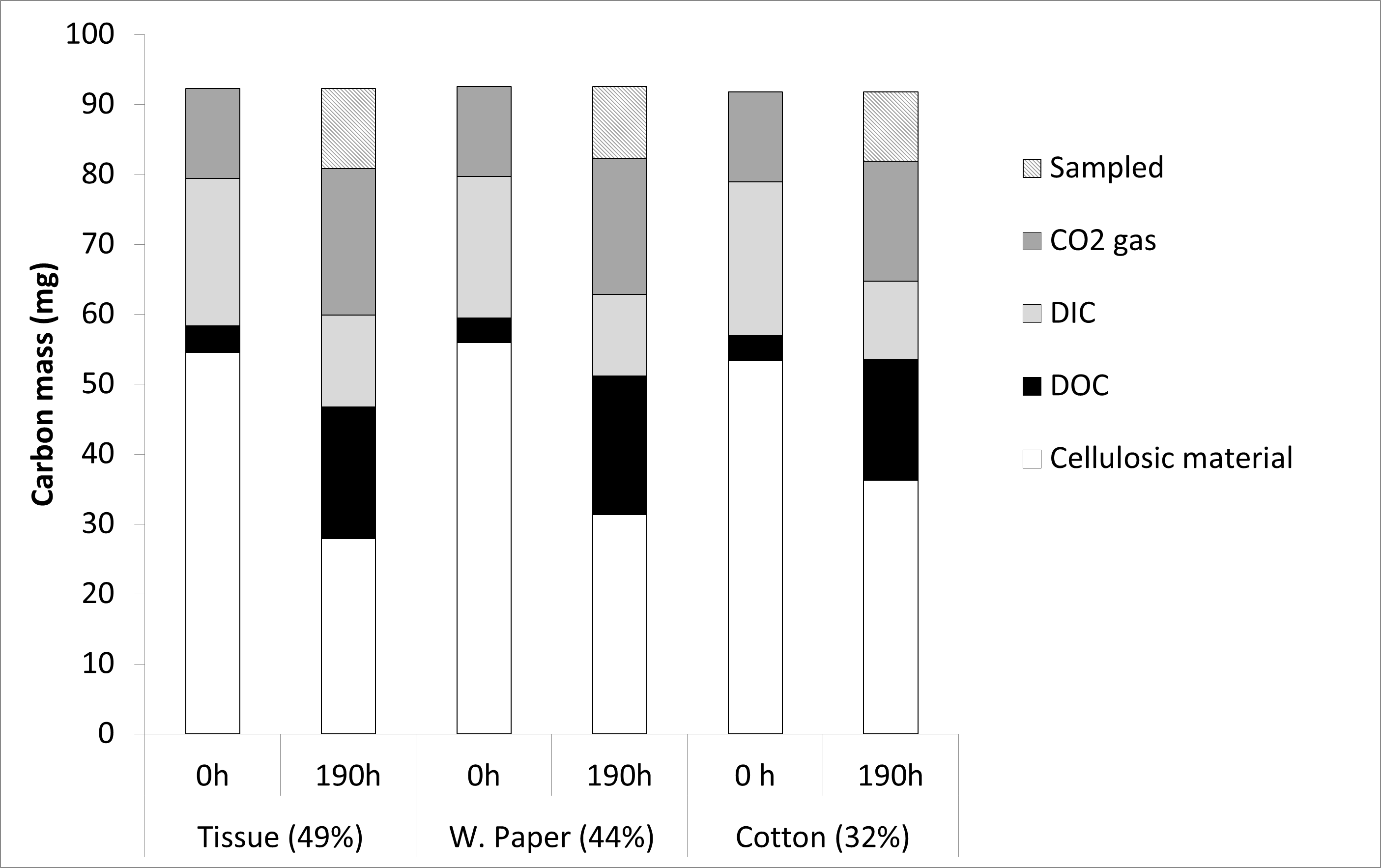

Supplement: S2 Fig — The carbon masses are in mg. Sampled: carbon mass removed from the microcosms through sampling of the liquid phase—CO2 gas: carbon mass in CO2 in the headspace—DIC: inorganic carbon mass in the liquid phase (Dissolved Inorganic Carbon)–DOC: organic carbon mass in the liquid phase (Dissolved Organic Carbon)–Cellulosic material: estimated carbon mass in the substrate (contained either in Tissue, Whatman Paper or Cotton). The percent values next to each substrate name indicate the estimated average degradation yield (percentage of carbon from the substrate that was degraded). Details on the calculation method are available in S1 File. (TIF) [file pone.0170524.s002.tif]

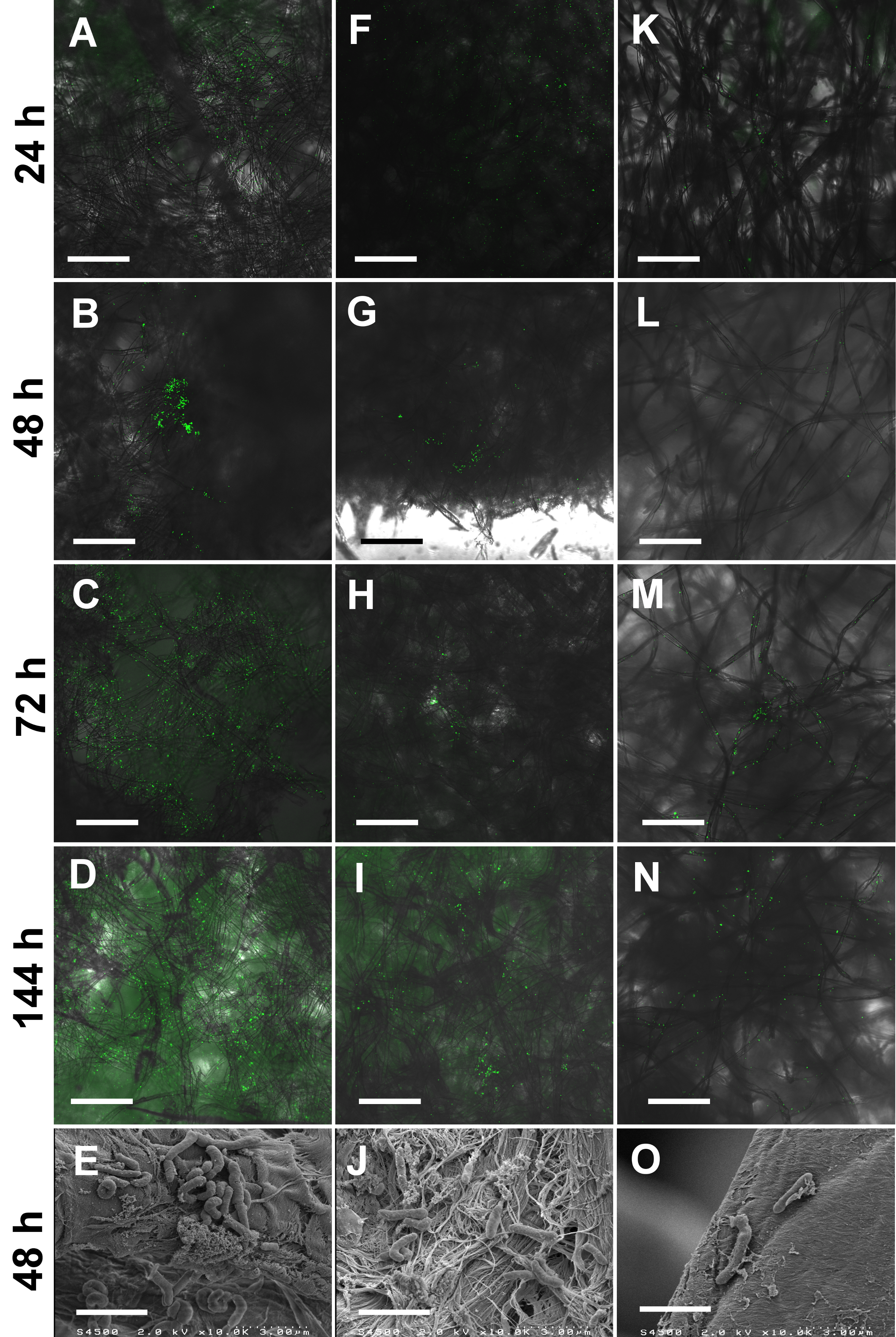

Supplement: S3 Fig — Confocal Laser Scanning Microscopy images were acquired from Tissue (A-D), Whatman Paper (F-I) and Cotton (K-N) incubations, on wet mount samples stained with a cellular esterase activity marker (green) after removal of the planktonic cells. Scale bars are 200 μm. A total of 75 representative images were acquired. Scanning Electron Microscopy images were acquired from Tissue (E), Whatman Paper (J) and Cotton (O) incubations, on samples collected after 48 h of incubation. Scale bars are 3 μm. A total of 137 images of scanning electron microscopic were acquired. Details on the methods are available in S1 File. (TIF) [file pone.0170524.s003.tif]

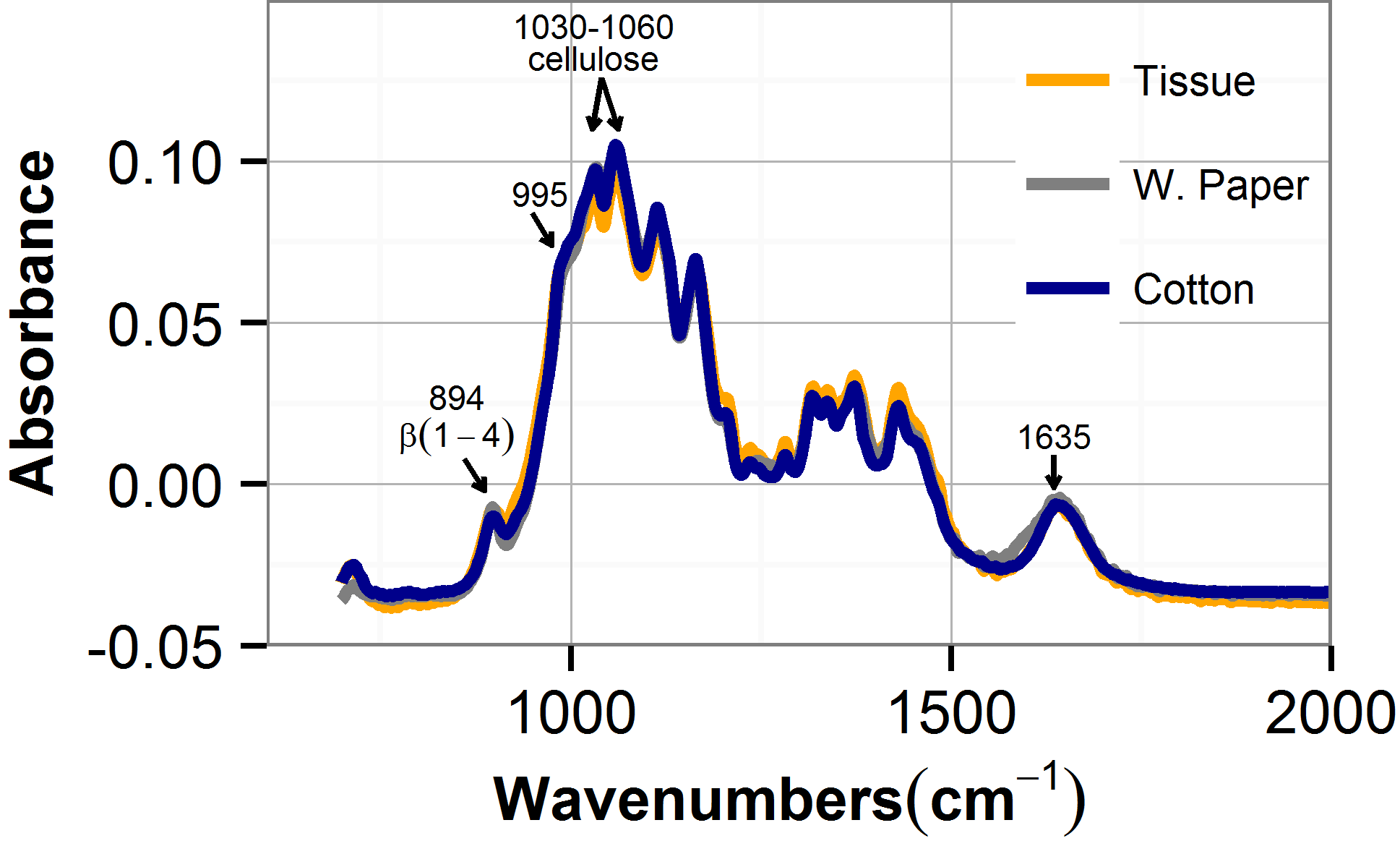

Supplement: S4 Fig — W. Paper: Whatman Paper. The 5 peaks annotated with arrows on the spectra are related to the presence of cellulose. Details on the method are available in S1 File. (TIFF) [file pone.0170524.s004.tiff]

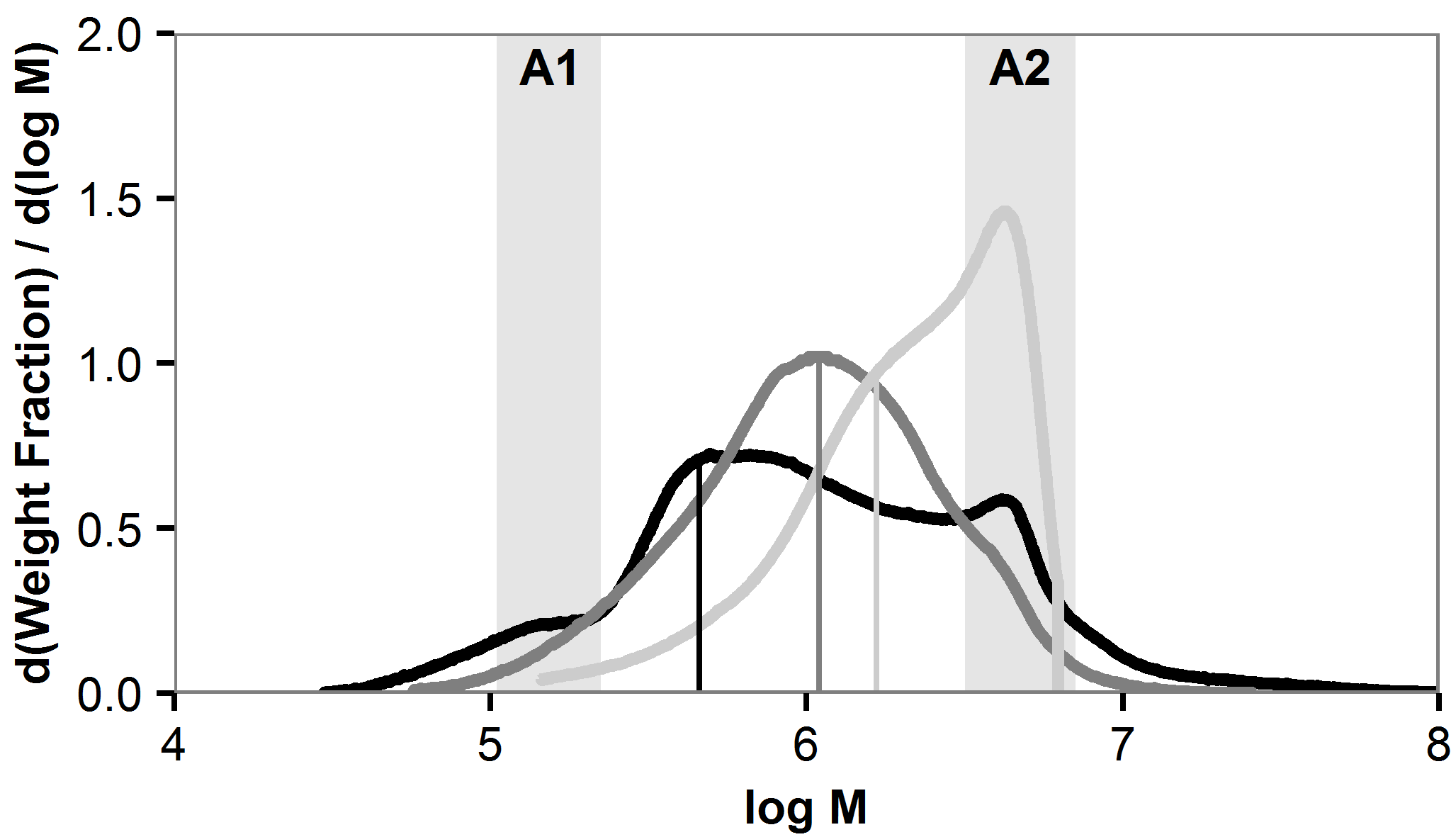

Supplement: S5 Fig — M stands for Molar Mass. Vertical colored lines indicate the positions of the M values corresponding to the peak of individual (i.e. non-aggregated) cellulose chains (see Table 1). Grey area A1: hemicellulose distribution peak observed for Tissue, originating from bleached wood pulp. Grey area A2: peaks corresponding to very high molecular weight polymers and, more likely, to cellulose chain aggregates. Details on the method are available in S1 File. (TIFF) [file pone.0170524.s005.tiff]

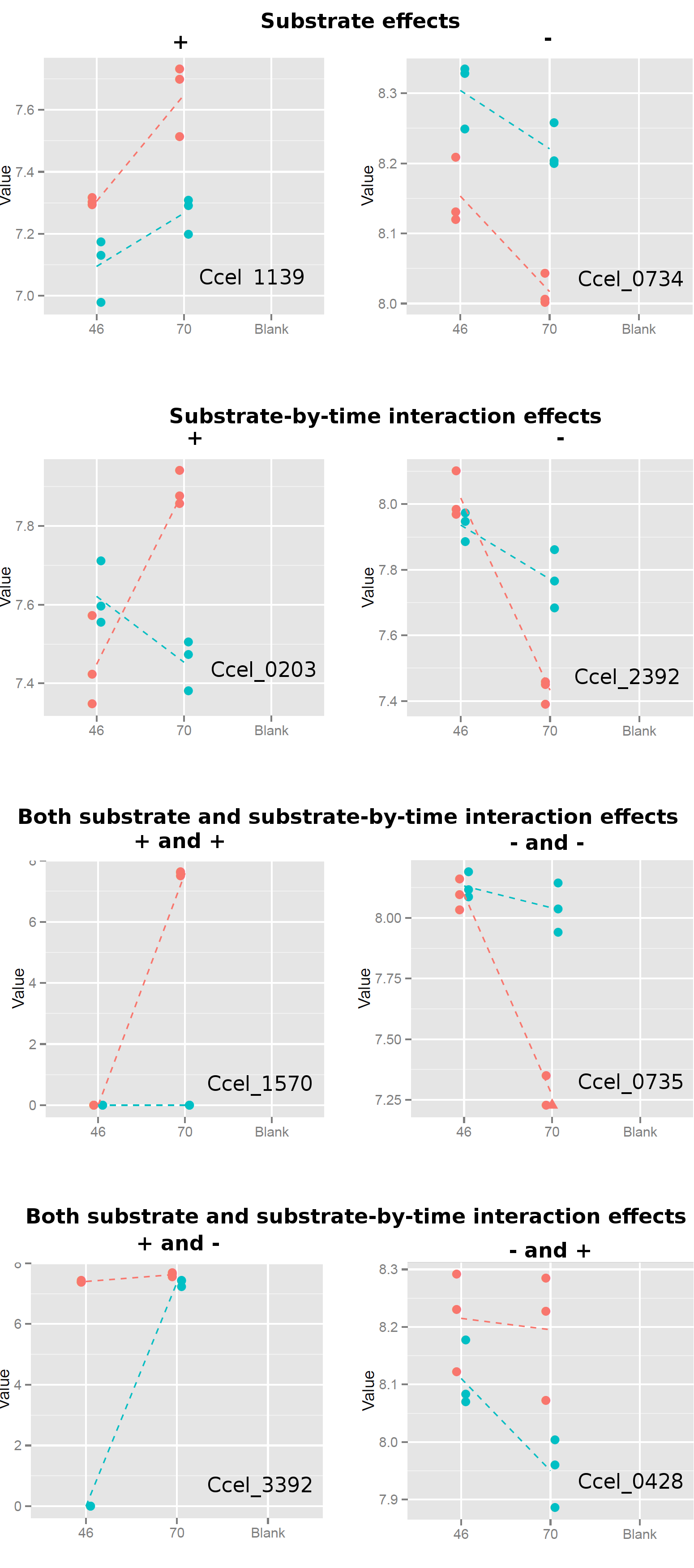

Supplement: S6 Fig — The illustrative examples are selected from the dataset of the pellet proteins. On each dot plot, the values are shown for the incubation times 46h, 70h and for the blank. The shown values correspond to the log-transformed and normalized data. The red color corresponds to Tissue incubations and the green color to Whatman Paper incubations. The “+” and “-”signs indicate the sign of the considered effect (substrate or substrate-by-time interaction). From left to right and from top to bottom: Ccel_1139 encodes a β-Glucosidase (see also Table 4); Ccel_0734 encodes the endoglucanase Cel9H (see also Table 2); Ccel_0203 codes for a β-Xylosidase (see also Table 4); Ccel_2392 codes for the endoglucanase/cellobiohydrolase Cel9V (see also Table 2); Ccel_1570 encodes a putative uncharacterized protein; Ccel_0735 encodes the endoglucanase Cel9J (see also Table 2); Ccel_3392 encodes a putative uncharacterized protein; Ccel_0428 encodes the endoglucanase Cel5I (see also Table 4). (TIF) [file pone.0170524.s006.tif]

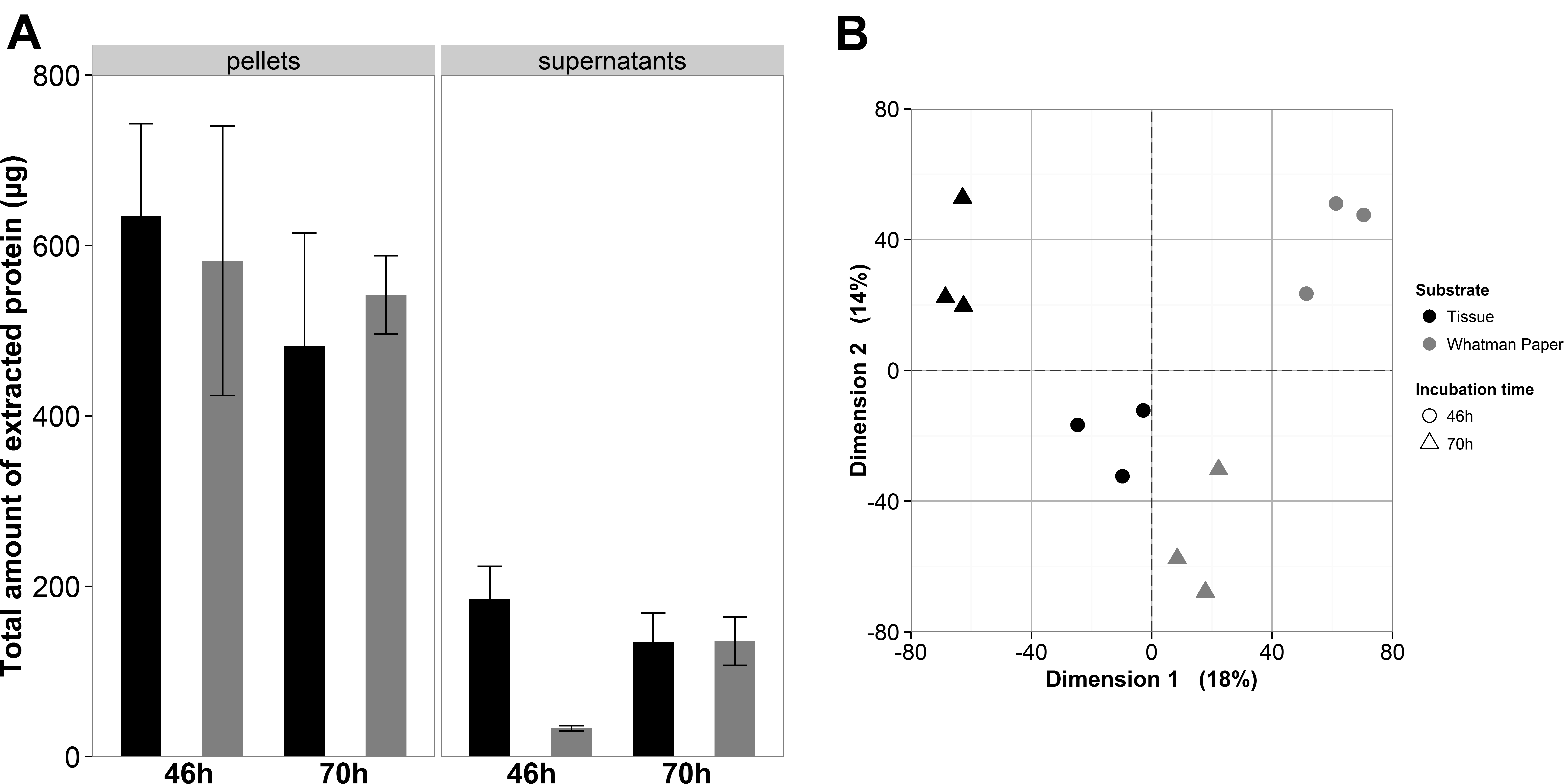

Supplement: S7 Fig — A) Total amounts of proteins extracted from the Tissue (black) and Whatman Paper (grey) incubations, after 46h and 70h of incubation, from the pellets and supernatants respectively. B) Principal component analysis of the samples based on the label-free quantitative proteomic data (XIC approach). (TIF) [file pone.0170524.s007.tif]

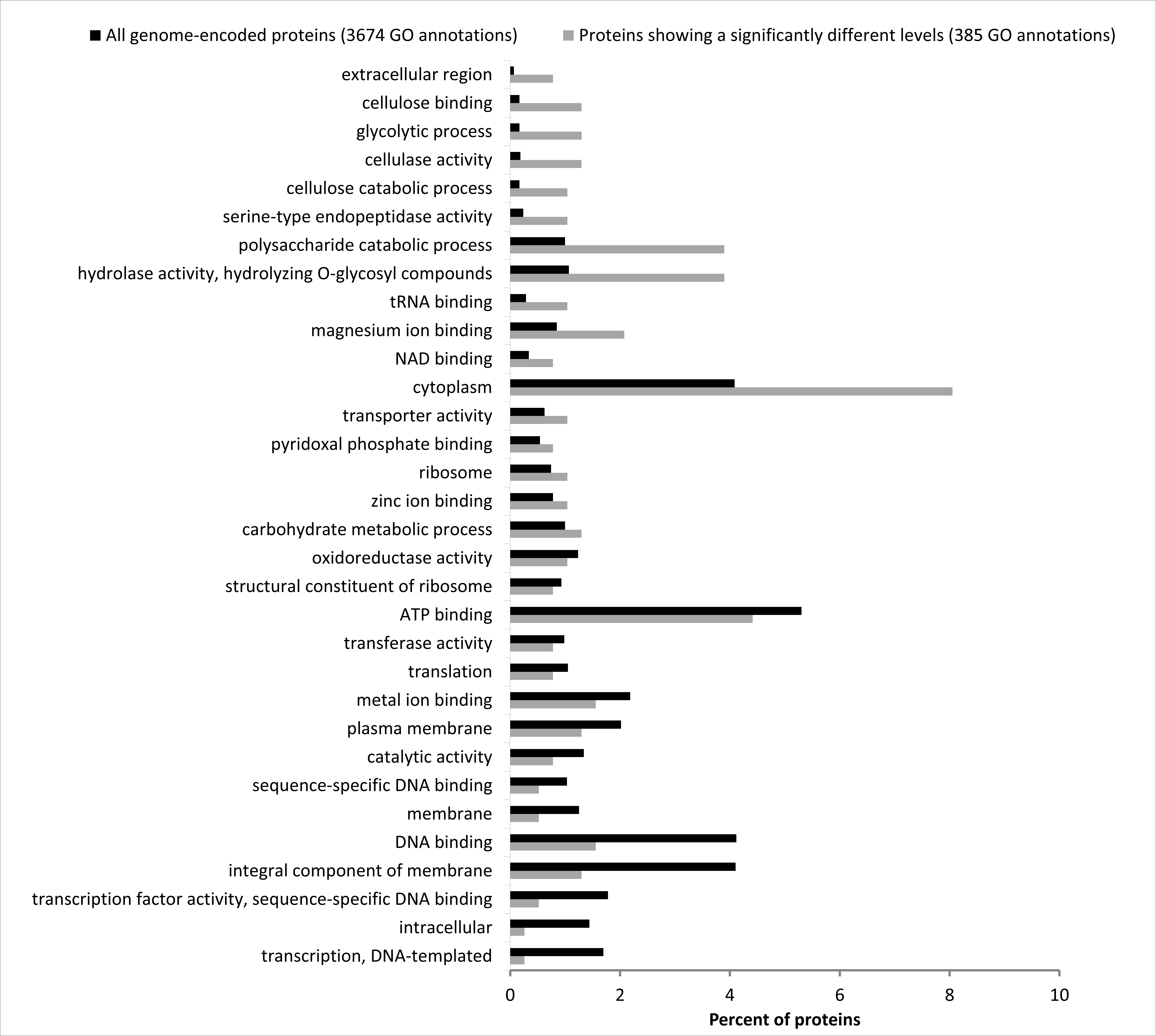

Supplement: S8 Fig — A selection of 32 Gene Ontology (GO) terms is shown, corresponding to the categories with highest percentages of annotations and to the most enriched or depleted categories when comparing the dataset of proteins with significantly different levels (after removal of categories with less than 3 proteins with significantly different levels) and all genome-encoded proteins. The GO terms are shown from the most enriched to the most depleted, from top to bottom. R. cellulolyticum genome encodes 3290 proteins, of which 2081 have GO annotations in UniprotKB, corresponding to a total of 3674 GO annotations. 151 proteins showed significantly different levels, of which 116 have GO annotations in UniprotKB, corresponding to a total of 385 annotations. Numeric values and additional details are shown in S2 Table. (TIF) [file pone.0170524.s008.tif]
