# Supplementary material for: Whole Proteome Analyses on Ruminiclostridium cellulolyticum Show a Modulation of the Cellulolysis Machinery in Response to Cellulosic Materials with Subtle Differences in Chemical and Structural Properties
Source: PLoS One. 2017 Jan 23;12(1):e0170524. doi: 10.1371/journal.pone.0170524 (PMC5256962; doi:10.1371/journal.pone.0170524)
Supplement: S1 Table — The carbon masses are in mg. The average values (± one standard deviation where relevant) are shown. Cellulosic material: carbon mass in the substrate (contained either in Tissue, Whatman Paper or Cotton)–DOC: organic carbon mass in the liquid phase (Dissolved Organic Carbon)–DIC: inorganic carbon mass in the liquid phase (Dissolved Inorganic Carbon)–CO2 gas: carbon mass in CO2 in the headspace—Sampled: carbon mass removed from the microcosms through sampling of the liquid phase—Total: total carbon mass in the microcosms at the initial incubation time point—Degradation yield: estimated percentage of degraded carbon from the substrate. The carbon mass in the cellulosic material at the final time point is calculated by considering that the total carbon mass is identical at time points 0h and 190h in the system. To calculate the carbon mass removed through sampling of the liquid phase, two options were considered: no substrate particles were sampled (option 1), substrate particles at a concentration of 2.6 g/L (corresponding to the initial concentration) were sampled (option 2). Consequently, two different values were obtained for the carbon mass in the cellulosic material at time point 190h. More details on the method are available in S1 File. (PDF) [file pone.0170524.s010.pdf]

	Tissue			Whatman Paper			Cotton		
	**0h**	**190h**		**0h**	**190h**		**0h**	**190h**	
Cellulosic material	54.5	27.9 ± 4.7	25.7 ± 4.7	56.0	31.3 ± 1.9	29.1 ± 1.9	53.4	36.2 ± 4.7	34.0 ± 4.7
DOC	3.8 ± 0.2	18.8 ± 2.6		3.5 ± 0.1	19.8 ± 1.1		3.5 ± 0.1	17.3 ± 2.7	
DIC	21.1 ± 0.8	13.2 ± 2.8		20.2 ± 1.3	11.7 ± 0.5		22.0 ± 0.7	11.2 ± 0.9	
CO_2 gas	12.9	20.9 ± 0.3		12.9	19.4 ± 0.4		12.9	17.1 ± 3.1	
Sampled (min / max)	-	11.5 ± 0.2	13.7 ± 0.2	-	10.3 ± 0.3	12.5 ± 0.3	-	9.9 ± 0.2	12.1 ± 0.2
**Total**	**92.3 ± 0.7**	-	-	**92.6 ± 1.3**	-	-	**91.8 ± 0.7**	-	-
**Degradation yield**	-	**48.8 ± 8.6 %**	**52.9 ± 8.6%**	**-**	**44.0 ± 3.3 %**	**48.0 ± 3.3 %**	**-**	**32.1 ± 8.8%**	**36.3 ± 8.8%**
