# Supplementary material for: Whole Proteome Analyses on Ruminiclostridium cellulolyticum Show a Modulation of the Cellulolysis Machinery in Response to Cellulosic Materials with Subtle Differences in Chemical and Structural Properties
Source: PLoS One. 2017 Jan 23;12(1):e0170524. doi: 10.1371/journal.pone.0170524 (PMC5256962; doi:10.1371/journal.pone.0170524)
Supplement: S1 File — (PDF) [file pone.0170524.s013.pdf]

Whole proteome analyses on *Ruminiclostridium cellulolyticum* show a modulation of the cellulolysis machinery in response to cellulosic materials with subtle differences in chemical and structural properties

Nelly Badalato, Alain Guillot, Victor Sabarly, Marc Dubois, Nina Pourette, Bruno Pontoire, Paul Robert, Arnaud Bridier, Véronique Monnet, Diana Z. Sousa, Sylvie Durand, Laurent Mazéas, Alain Buléon, Théodore Bouchez, Gérard Mortha, Ariane Bize

Supplementary Materials and Methods

Shotgun MS/MS analyses

5 µg of purified proteins were fixed in SDS-PAGE gel (NuPAGE Bis-Tris Mini Gels, Invitrogen). After a short-duration migration, gel fragments (~5 mm) containing the proteins were excised. In-gel digestion was performed with the Progest system (Genomic Solution) according to a standard trypsinolysis protocol [3]. Liquid chromatography coupled to tandem mass spectrometry (LC-MS/MS) analyses were performed on an Ultimate 3000 RSLCnano system (Dionex, Voisins le Bretonneux, France) connected to a LTQ-Orbitrap Discovery mass spectrometer (Thermo Fisher, USA) *via* a nanoelectrospray ion source. Tryptic peptide mixtures (4 µl) were loaded at 20 µl/min flow rate onto a desalting precolumn Pepmap C18 (0.3 x 5 mm, 100 Å, 5 µm, Dionex). After 4 min, the precolumn was connected to the separating nanocolumn Pepmap C18 (0.075 x 50 cm, 100 Å, 2 µm) and the peptides were eluted with a two-step gradient of buffer B (80 % acetonitrile, 0.1 % formic acid) in buffer A (2 % acetonitrile, 0.1 % formic acid), from 1 % to 30 % during 162 min and 30-40 % during 10 min at a flow rate of 300 nl/min for a total acquisition time of 188 min. Ionization was performed on liquid junction with a spray voltage of 1.4 kV applied to non-coated capillary probe (PicoTip EMITER 10-µm tip inner diameter, New Objective, USA). Peptide ions were automatically analyzed by the data-dependent method in Xcalibur software (Thermo Scientific, version 2.0.7) with the following acquisition steps: (1) full scan MS (*m/z* 300-1400) in the Orbitrap analyzer with a resolution of 15000 and (2) MS/MS (fragmentation and detection) on the 10 most abundant precursors in the LTQ linear ion trap. Only the doubly and triply charged precursor ions were subjected to MS/MS fragmentation with classical peptide fragmentation parameters (Qz = 0.22, activation time = 50 ms, collision energy = 35 %, isolation window of 2 th, dynamic exclusion time of 60 s).

Degradation yield

To estimate the degradation yield, carbon mass distributions were calculated at the initial and final incubation time points (0h and 190h respectively). The mass of cellulosic material was not measured during the incubation and it was thus estimated by considering that the total amount of carbon in the system is identical at both time points. For the cellulosic material at time point 0h, the amount of introduced substrate is considered as known and identical for all microcosms. For the DOC (dissolved organic carbon) and DIC, the carbon masses were calculated directly from the measured experimental values. For CO_2 gas, the amount of introduced gas is considered as known and identical for all microcosms; the carbon masses at the final time points were calculated directly from measured experimental values (microGC analyses). For the carbon masses removed through sampling of the liquid phase, two different options were considered: first, that no substrate particles were removed during the sampling; second, that substrate particles at a concentration of 2.6 g/L (corresponding to the initial concentration) were removed. A minimal and a maximal values were thus obtained for the removed carbon mass, resulting in two different values for the carbon mass in the substrate at the final incubation time point. The carbon contained in the biomass was estimated from the extracted DNA amounts. It was negligible compared to other values and is thus not shown.

Imaging

For imaging, reactors consisted in 96-well microplates incubated in sealed anaerobic bags with 300 µl working volume in each well (same minimal medium). For each different substrate, chads were punched and added to 5 wells, resulting in a final substrate concentration of approximately 5 g/L. At each sampling time point, one microplate was sacrificed and the samples were prepared for imaging. The liquid phase was removed from each well and cellulose fibers were washed with 1X phosphate buffer saline (PBS). For live cell imaging, 4 wells were prepared for each substrate as follows: cells were stained with 0.1 % ChemChrome V8 (Chemunex), a cellular esterase activity marker, in 1X PBS for 10 min at 37°C. Observations were realized directly after staining using a confocal laser scanning microscope (LSM 510 Zeiss). For scanning electron microscopy, 1 well was prepared for each substrate as follows: samples were fixed in a solution containing 2.5 % glutaraldehyde and 0.1 M sodium cacodylate (pH 7.4) for 16 h and stored at 4°C until further analyses. Before observation, samples were prepared as described in [4]. The observations were performed at the INRA platform MIMA2 (INRA, Massy, France, http://www6.jouy.inra.fr/mima2/).

Fourier transform infrared spectroscopy (FTIR)

For FTIR by mid-infrared spectroscopy, 2 mg of each material (Tissue, Cotton, Whatman Paper) was mixed with 120 mg of spectroscopically pure potassium bromide and measurements were realized as described in [5]. The spectral region between 2000 and 700 cm^{-1} was treated using the OPUS software (v 2.06) as indicated before analysis.

Principal component analyses of the proteomes

The protein corresponding to contaminants were excluded from the analysis. For each culture sample, the data obtained for the cell pellet and the supernatant were combined. The analysis was subsequently performed after log10-transformation and quantile normalization of the data, using the R CRAN package FactoMineR [6] on unscaled data. The percentage of variance associated to the considered dimension is indicated on each axis. The samples from the Whatman Paper incubations at 70 h and from the Tissue incubations at 46 h appear as the most similar, consistent with fermentation occurring faster in the Tissue than in the Whatman Paper incubations.

Supplementary References

1. Xiao B, Sun X, Sun R (2001) Chemical, structural, and thermal characterizations of alkali-soluble lignins and hemicelluloses, and cellulose from maize stems, rye straw, and rice straw. Polymer Degradation and Stability 74: 307-319.
2. Fengel D, Wegener G (1983) Wood: chemistry, ultrastructure, reactions: Walter de Gruyter.
3. Shevchenko A, Tomas H, Havli, sbreve J, Olsen JV, et al. (2007) In-gel digestion for mass spectrometric characterization of proteins and proteomes. Nature protocols 1: 2856-2860.
4. Bridier A, Meylheuc T, Briandet R (2013) Realistic representation of Bacillus subtilis biofilms architecture using combined microscopy (CLSM, ESEM and FESEM). Micron 48: 65-69.
5. Devaux MF, Barakat A, Robert P, Bouchet B, Guillon F, et al. (2005) Mechanical breakdown and cell wall structure of mealy tomato pericarp tissue. Postharvest biology and technology 37: 209-221.
6. Husson F, Pagès J, Lê S (2010) Exploratory multivariate analysis by example using R. AMC 10: 12.
